# Intrageneric cross-reactivity of monospecific rabbit antisera against venoms of the medically most important *Bitis* spp. and *Echis* spp. African snakes

**DOI:** 10.1371/journal.pntd.0010643

**Published:** 2022-08-12

**Authors:** Aarón Gómez, Andrés Sánchez, Gina Durán, Daniel Cordero, Álvaro Segura, Mariángela Vargas, Daniela Solano, María Herrera, Stephanie Chaves-Araya, Mauren Villalta, Melvin Sánchez, Mauricio Arguedas, Cecilia Díaz, José María Gutiérrez, Guillermo León

**Affiliations:** Instituto Clodomiro Picado, Facultad de Microbiología, Universidad de Costa Rica, San José, Costa Rica; Liverpool School of Tropical Medicine, UNITED KINGDOM

## Abstract

**Background:**

Snakebite envenomation exerts a heavy toll in sub-Saharan Africa. The design and production of effective polyspecific antivenoms for this region demand a better understanding of the immunological characteristics of the different venoms from the most medically important snakes, to select the most appropriate venom combinations for generating antivenoms of wide neutralizing scope. *Bitis* spp. and *Echis* spp. represent the most important viperid snake genera in Africa.

**Methodology/Principal findings:**

Eight rabbit-derived monospecific antisera were raised against the venoms of four species of *Bitis* spp. and four species of *Echis* spp. The effects of immunization in the rabbits were assessed, as well as the development of antibody titers, as judged by immunochemical assays and neutralization of lethal, hemorrhagic, and *in vitro* coagulant effects. At the end of immunizations, local and pulmonary hemorrhage, together with slight increments in the plasma activity of creatine kinase (CK), were observed owing to the action of hemorrhagic and myotoxic venom components. Immunologic analyses revealed a considerable extent of cross-reactivity of monospecific antisera against heterologous venoms within each genus, although some antisera provided a more extensive cross-reactivity than others. The venoms that generated antisera with the broadest coverage were those of *Bitis gabonica* and *B*. *rhinoceros* within *Bitis* spp. and *Echis leucogaster* within *Echis* spp.

**Conclusions/Significance:**

The methodology followed in this study provides a rational basis for the selection of the best combination of venoms for generating antivenoms of high cross-reactivity against viperid venoms in sub-Saharan Africa. Results suggest that the venoms of *B*. *gabonica*, *B*. *rhinoceros*, and *E*. *leucogaster* generate antisera with the broadest cross-reactivity within their genera. These experimental results in rabbits need to be translated to large animals used in antivenom production to assess whether these predictions are reproduced in horses or sheep.

## Author summary

Snakebite envenomation exerts a heavy toll in sub-Saharan Africa. Antivenom is the only valid therapy for these envenomations. However, owing to the wide variety of snake species and the large variation in venom composition, the selection of the best combination of venoms to generate effective polyclonal antivenoms of wider neutralizing coverage is challenging. In this study, an experimental protocol was developed based on the generation of monospecific antisera in rabbits immunized with venoms of *Bitis* spp. or *Echis* spp. from Africa. Cross-reactivity of monospecific antisera was assessed by immunochemical analyses and by neutralization of toxic effects. Results revealed a large extent of intrageneric cross-reactivity by all antisera, with few exceptions. The venoms that generated antisera with the highest cross-reactivity and neutralizing ability among *Bitis* spp. were those of *Bitis gabonica* and *B*. *rhinoceros*, and of *Echis leucogaster* in *Echis* spp. These results provide evidence for the selection of the best combination of venoms for preparing antivenoms of high neutralizing scope within these two medically relevant genera of African viperid species. These results in rabbits must be translated to large animals used in antivenom production to assess whether these predictions are confirmed.

## Introduction

Snakebite envenomation is an important public health problem in tropical and sub-tropical countries around the world, especially in impoverished rural agricultural and pastoral communities in sub-Saharan Africa, Asia, Latin America, and some regions of Oceania [1, 2]. Therefore, the World Health Organization considers it as a highest impact (i.e., Category A) neglected tropical disease [1, 3].

It has been estimated that 1.2–5.5 million of snakebites occurred worldwide in 2007, from which 421,000–1,841,000 cases resulted in envenomation, and 20,000–94,000 in death. Approximately 27% of these incidents occurred in Africa [4]. According to the reptile database (https://reptile-database.reptarium.cz/), there are 61 species of snakes of the family Viperidae in Africa, which are grouped in five genera (i.e., *Atheris*, *Bitis*, *Causus*, *Cerastes*, and *Echis*). The species with major potential to induce envenomations of high incidence and severity belong to the *Bitis* and *Echis* genera.

Among the 14 African *Bitis* species, only *B*. *arietans* (Puff adder), *B*. *gabonica* (East African Gaboon viper), *B*. *nasicornis* (Rhinoceros viper), and *B*. *rhinoceros* (West African Gaboon viper) are considered by WHO as highly venomous snakes whose bites result in high levels of morbidity, disability, or mortality (i.e., Category 1 species) [1]. On the other hand, all five species of African *Echis*, i.e., *E*. *coloratus* (Palestine saw-scaled viper), *E*. *jogeri* (Mali carpet viper), *E*. *leucogaster* (White-bellied carpet viper), *E*. *ocellatus/romani* (West African carpet viper), and *E*. *pyramidum* (North-East African carpet viper), are included in the Category 1 in the WHO classification [1].

Venoms of *Bitis* spp. and *Echis* spp. are composed of different proportions of proteins belonging to the same families, such as Zn^2+^-dependent snake venom metalloproteinases (SVMPs), snake venom serine proteinases (SVSPs), phospholipases A_2_ (PLA_2_s), cysteine-rich secretory proteins (CRISPs), C-type lectin-like proteins, disintegrins, L-amino acid oxidases (LAAOs), Kunitz-type inhibitors, and other less abundant components [5–9].

Envenomations by species of *Bitis* and *Echis* can be differentiated according to their clinical manifestations: cases produced by *Bitis* spp. are characterized by marked local swelling with coagulable blood, with the exceptions of some populations of *B*. *arietans* which induce coagulopathies [10, 11]. On the other hand, the syndrome induced by *Echis* spp. is characterized by marked local swelling, with incoagulable blood and/or spontaneous systemic bleeding [11]. In addition, and depending on the severity of the envenomation, clinical manifestations could include local necrosis, blistering, and hemodynamic disturbances, among other effects [12].

Intravenous administration of animal-derived antivenoms is the only validated therapy for snakebite envenomations [1]. Antivenoms are formulations of whole immunoglobulins, or their Fab or F(ab´)_2_ fragments, purified from plasma of animals immunized towards snake venoms [1, 13]. Depending on the number of venoms used for immunization, antivenoms can be either monospecific, bispecific or polyspecific.

The anti-venom immunoglobulins bind and neutralize venom toxins in the circulation or tissues and contribute to their elimination. However, their efficacy is limited to venoms antigenically similar to those used as immunogens to stimulate the immune response in animals. Therefore, to ensure a wide coverage of neutralization without requiring the identification of the offending snake species, the use of polyspecific formulations is generally preferred [1, 14]. This is the case of antivenoms designed for use in sub-Saharan Africa, most of which are polyspecific, although some monospecific products are also available [11].

To produce polyspecific antivenoms with wide neutralization scope, venoms used as immunogens should be selected considering the intra- and inter-specific variation in their composition, and the intrageneric conservation of the antigenic characteristics of venoms of the medically most important snakes in the region where the antivenom is intended to be used [1]. The selection of the most appropriate venoms for immunization should therefore be based on a detailed knowledge of the antigenic relatedness of venoms and the medical relevance of the species. This represents a particular challenge in the case of Africa, where abundant venomous snakes are widely distributed [1].

In this work, we inferred the antigenic similarity between venoms of *Bitis* spp. and *Echis* spp. classified by the WHO as African Category 1 snakes on the basis of the intrageneric cross-reactivity between monospecific rabbit sera raised against individual venoms. This information is of value for the rational, knowledge-based design of the most appropriate venom mixtures for the generation of pan-African antivenoms.

## Materials and methods

### Ethics statement

This manuscript presents an experimental study performed following the standard procedures of scientific ethics, including those related to the use and care of animals. All procedures carried out in this study meet the International Guiding Principles for Biomedical Research Involving Animals [15]. All procedures involving animals were approved by the Institutional Committee for the Care and Use of Laboratory Animals of Universidad de Costa Rica (approval code CICUA 202–2020). Mice and rabbits were obtained from the Bioterium of Instituto Clodomiro Picado. Mice were handled in Tecniplast Eurostandard Type II 1264C cages (L25.0 x W40.0 x H 14.0 cm), five mice per cage, while rabbits were managed in Scanbur type EC3 cages (L 823 x W 660 x H 110 mm), one rabbit per cage. In both cases, animals were maintained at 18–24°C, 60–65% relative humidity and 12:12 light-dark cycle. Human plasma for coagulation experiments was collected from healthy donors who were over 18 years old and provided written informed consent, according to the normative of the Institutional Bioethics Committee of Universidad de Costa Rica (approval VI-2925-2013).

### Snake venoms

Venoms of adult specimens of *B*. *arietans* (unspecified origin, batch #322.061), *B*. *gabonica* (unspecified origin, batch #725.031), *B*. *nasicornis* (unspecified origin, batch #500.102), *B*. *rhinoceros* (from Ghana, batch #701.070), *E*. *coloratus* (from Egypt, batch #512.191), *E*. *leucogaster* (from Mali, batch #623.070), *E*. *ocellatus* (unspecified origin, batch #216.031), and *E*. *pyramidum* (from Egypt, batch #523.070) were purchased from Latoxan (Portes-dès Valence, France). After collection, venoms were stabilized by lyophilization and stored at -40°C. Solutions of venoms were prepared immediately before use.

### Reverse-phase HPLC profiling

Five milligrams of each venom were dissolved in 200 μL of 0.1% trifluoroacetic acid (TFA) and 5% acetonitrile buffer (buffer A). Insoluble material was removed by centrifugation and the proteins in the supernatant were separated by reverse-phase HPLC (RP-HPLC, HPLC system: Agilent 1100 series; Agilent Technologies), equipped with a C18 column (250 x 4.6 mm, 5 μm particle size; Agilent Technologies). The flow rate was set to 1 mL/min and the protein separation was performed with the following buffer gradient: 0% buffer B (buffer B: 95% acetonitrile, 0.1% TFA) for 5 min, followed by 0–15% B over 10 min, 15–45% B over 60 min, 45–70% B over 10 min and 70% B for 9 min [16]. Protein peaks were detected at 215 nm. HPLC fractions were identified by comparing the chromatograms with those previously published for *B*. *arietans*) [17], *B*. *gabonica* [6], *B*. *nasicornis* and *B*. *rhinoceros* [7], and *E*. *ocellatus* [5]. We did not find published proteomic analysis of venoms of *E*. *coloratus*, *E*. *leucogaster* and *E*. *pyramidum* that could be used as a reference.

### Determination of toxic activities of venoms

#### Lethal activity

Groups of five mice (16–18 g; CD-1 strain; both sexes) received a subcutaneous (SC) injection of the analgesic Tramadol, at a dose of 50 mg/kg, to reduce pain during the tests [18]. Fifteen minutes afterwards, mice received an intraperitoneal (IP) injection of 0.5 mL of 0.12 M NaCl, 0.04 M phosphate, pH 7.2 solution (PBS) containing different amounts of venoms (12.0–91.1 μg/mouse for venoms of *Bitis* spp, or 10.0–113.9 μg/mouse for venoms of *Echis* spp). The number of deaths during the following 6 h was recorded [19] and used to estimate the median lethal dose (LD_50_, i.e., the amount of venom that results in the death of 50% of the injected mice) by Probits [20]. Surviving mice were euthanized by CO_2_ inhalation. Results were reported as LD_50_ and the corresponding 95% confidence interval (95% CI).

### Hemorrhagic activity

Groups of three mice (18–20 g; CD-1 strain; both sexes) were treated by the SC route with the analgesic Tramadol, at a dose of 50 mg/kg. Fifteen minutes afterwards, mice received an intradermal (ID) injection of various doses of venoms (0.05–0.80 μg/mouse for venoms of *Bitis* spp, or 0.10–3.20 μg/mouse for venoms of *Echis* spp), dissolved in 100 μL PBS. After 2 h, mice were sacrificed by CO_2_ inhalation. Then, by using the Inkscape 0.91 program (https://inkscape.org/download/), the area and intensity of the hemorrhagic lesion induced by the venom were measured in the inner side of the skin and expressed in hemorrhagic units (HaU). The minimum hemorrhagic dose (MHD) was defined as the amount of venom that generates 100 HaU [21]. Results were reported as the mean ± SD (n = 3).

### Coagulant activity *in vitro*

Coagulant activity of venoms was determined based on a turbidimetric assay [22, 23]. Different amounts of venoms, dissolved in 100 μL of 25 mM Tris-HCl, 137 mM NaCl, 3.4 mM KCl, pH 7.4 (TBS) were added in triplicate to wells in a 96-well plate and incubated for 5 min at 37°C in a microplate reader (Cytation 3 Imaging Reader, Bio- Tek). Then, 4 μL of 0.4 M CaCl_2_ was added to 100 μL of human citrated plasma previously incubated at 37°C, and this mixture was added immediately to each venom-containing well using a multichannel pipette. Samples were mixed for 5 s by shaking, and the absorbance at 340 nm was monitored every 30 s over 15 min. The increase in absorbance reflects the formation of a clot. The minimum coagulant dose (MCD) was calculated as the amount of venom that induce a change in absorbance of 0.01 units within 1 min. Results were reported as the mean ± SD of triplicate determinations.

### Immunization of rabbits

Groups of four rabbits (New Zealand, 2.5–3.0 kg body weight) were immunized with the venoms of either *B*. *arietans*, *B*. *gabonica*, *B*. *nasicornis*, *B*. *rhinoceros*, *E*. *coloratus*, *E*. *leucogaster*, *E*. *ocellatus*, or *E*. *pyramidum*. Immunization was carried out by five SC injections, applied at two-week intervals. The total volume of injections was 2 mL and each one of them contained 1 mg of venom. In the first injection, the venoms were emulsified in Complete Freund’s Adjuvant (CFA). In the second one, Incomplete Freund’s Adjuvant (IFA) was used, and in the following injections venoms were dissolved in PBS. Samples of blood were collected from the ear marginal vein, at the end of the immunization, for hematological, serum chemistry, and immunological analyses. Rabbits were then euthanized by an overdose of anesthetic (i.e., a dose of 100 mg/kg of sodium pentobarbital, administered by the IP route). A group of four non-immunized rabbits was included in parallel with immunized animals, and used as control for the hematological, serum chemistry, necropsy, and immunological analyses.

### Hematological, serum chemistry and necropsy analysis

Hematological, serum chemistry, and necropsy analyses were applied to each individual rabbit sample. Hematological analyses (i.e., erythrocytes, leukocytes and platelets count, hematocrit, and hemoglobin concentration) were carried out in a Veterinary Hematology Analyzer (Exigo Eos Hematology System; Boule Diagnostics AB, Stockholm, Sweden). The following analytes were quantified in a clinical chemistry analyzer (Spin200E Automatic biochemistry analyzer; Spinreact, Barcelona, España): creatine kinase (CK), alanine aminotransferase (ALT), aspartate aminotransferase (AST), and alkaline phosphatase (ALP), and were determined by the corresponding International Federation of Clinical Chemistry and Laboratory Medicine (IFCC) methods. Urea was quantified by a modification of the Talke and Schubert method [24]; creatinine by a kinetic modification of the Jaffe colorimetric method [25]; albumin by the bromocresol green colorimetric method [26]; and total protein by the Biuret method [27]. Immediately after euthanasia, tissue samples of skin, liver, lungs, kidneys, and heart, were collected and added to 3.7% formalin solution. Tissues were processed routinely and embedded in paraffin. Sections of 4 μm thickness were obtained and stained with hematoxylin-eosin for histological analysis.

### Reactivity of rabbit sera by enzyme-linked immunosorbent assay (ELISA)

Polystyrene plates were coated overnight at room temperature with 100 μL of PBS containing 3 μg of venom. After washing the plates five times with distilled water, 100 μL of several dilutions of each rabbit serum sample, in PBS-2% bovine serum albumin (BSA), were added. Plates were incubated for 1 h at room temperature and washed five times. Afterwards, 100 μL of goat anti-rabbit IgG conjugated with peroxidase, diluted 1:5000 with PBS-2% BSA, were added to each well. Microplates were incubated for 1 h at room temperature. After a final washing step, color was developed by the addition of H_2_O_2_ and *o*-phenylenediamine, during 20 min. Color development was stopped by the addition of 1.0 M HCl. Absorbances at 492 nm were recorded. The relative concentration of anti-venom antibodies in the samples was calculated by interpolation of their absorbances in a calibration curve. Relative concentration was expressed as percentage, 100% corresponding to the titer of the serum raised against the homologous venom of each species. Results were expressed as mean ± SD of all rabbits in each group. Heterologous sera with content of specific antibodies higher than 33% of the corresponding homologous serum were arbitrarily considered as “ELISA cross-reactive sera” and were selected for the assessment of *in vivo* neutralization assays.

### Electrophoretic analysis and western blot

SDS-PAGE was run under reducing conditions using an acrylamide concentration of 12% [28]. Gels were stained with Coomassie Brilliant Blue R-250 or transferred to a nitrocellulose membrane at 30 mAmp overnight. Then, the membranes were blocked with PBS-0.1% casein for 30 min. Next, membranes were incubated for 1 h with a pool of serum samples of all rabbits of each monospecific antiserum, diluted 1/500 with PBS-0.1% casein. After washing the membranes three times with PBS-0.1% casein, they were incubated for 1 h with goat anti-rabbit IgG conjugated with alkaline phosphatase, diluted 1:2000 with PBS-0.1% casein. Finally, after the last washing step, 5-bromo-4-chloro-3-indolyl-phosphate/nitroblue tetrazolium (BCIP/NBT) color development substrate was added, and the reaction was stopped, after 20 min, with distilled water.

### Neutralization of the lethal, hemorrhagic, and coagulant activities

To reduce the number of mice used in the *in vivo* neutralization assays, only the antisera that gave higher than 33% cross-reactivity by ELISA were evaluated in the *in vivo* assays. The ability of pools of serum samples from all rabbits in each group to neutralize the activities of the venoms was assessed by mixing a constant challenge dose of each venom with different dilutions of the pool of each antiserum. Mixtures were incubated at 37°C for 30 min before determining the residual activity of venom by using the experimental systems described above. Five and three mice per group were used in the neutralization assays of lethality and hemorrhage, respectively. The challenge doses utilized were: 2 LD_50_s for lethal activity, 5 MHDs for hemorrhagic activity and 2 MCDs for coagulant activity. In all cases, venom-only controls were included, in which venoms were incubated with PBS instead of antiserum. The use of 2 LD_50_s as a challenge dose in the neutralization of lethality studies, instead of the usual 4–5 LD_50_s, is justified to increase the sensitivity of the assay, i.e., to optimize the detection of cross-reactivity of antisera against venoms. For lethal and hemorrhagic activities, neutralization was expressed as the median effective dose (ED_50_), defined as the ratio mg venom/mL antiserum at which the activity of venom was reduced to 50% [29]. For coagulant activity, neutralization was expressed as Effective Dose (ED), defined as the ratio of venom/antivenom in which the change in absorbance is prolonged three times as compared to plasma incubated with venom alone [23].

### Statistical analysis

In the case of lethality and its neutralization, groups having non-overlapping values of 95% CI were considered significantly different. For the other toxic effects and ELISA, the significance of the differences between mean values of groups was assessed by one-way ANOVA, followed by a Ryan-Einot-Gabriel-Welsch Range (R-E-G-W Q) and Dunnett’s t post-hoc test, when applicable. Regarding specific comparisons between antisera and venoms in hemorrhagic and coagulant activities, a *t* test was performed. Linearity and homogeneity of variances were tested, and a p-value <0.05 was considered significant.

## Results and discussion

### General characterization of the *Bitis* spp. and *Echis* spp. venoms

**Fig 1:**
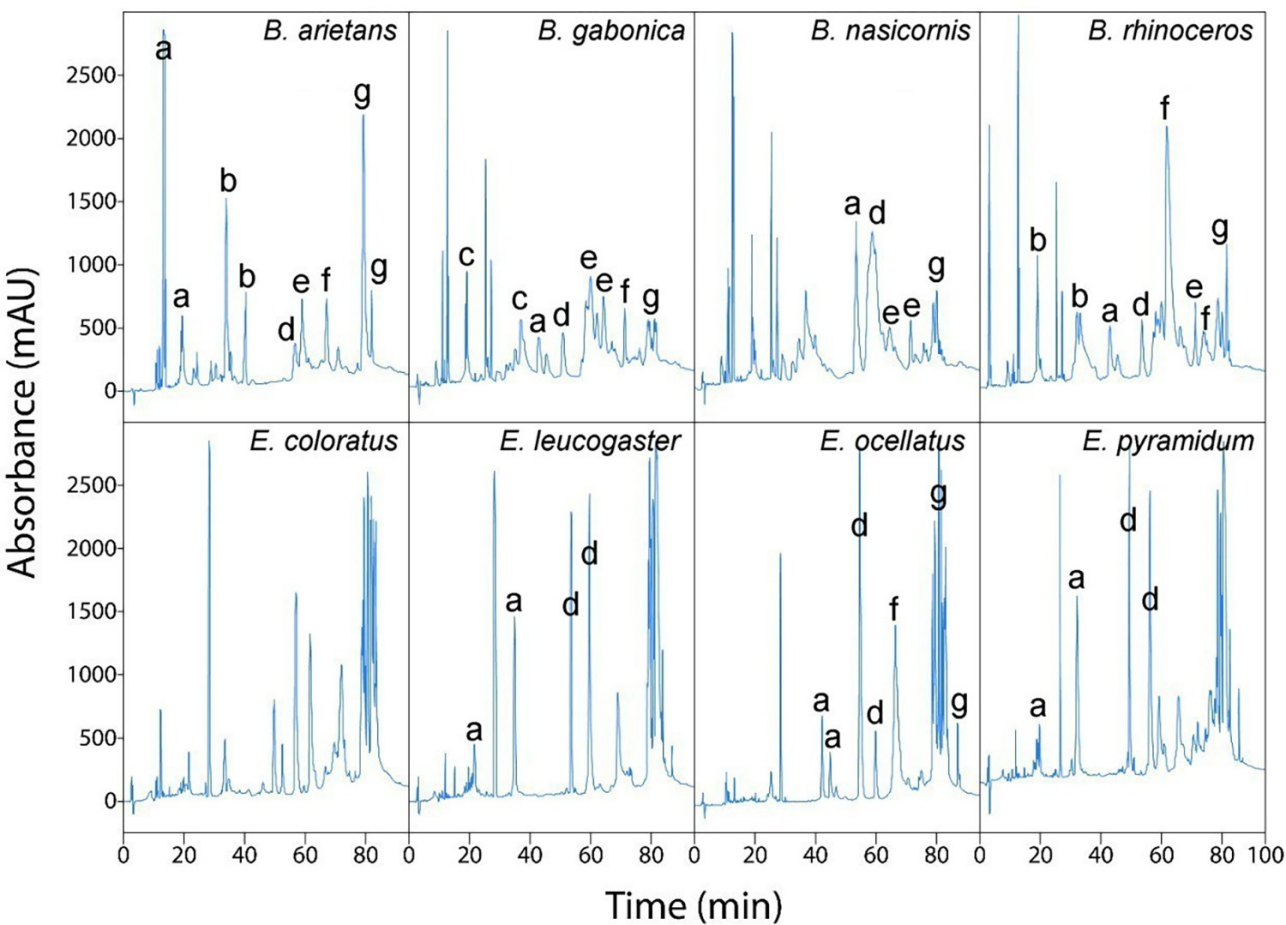
RP-HPLC chromatograms of venoms. Families of the most abundant toxins in the venom fractions were identified according to previously published proteomic analyses: a) disintegrins, b) Kunitz-type inhibitors, c) bradykinin potentiating peptides, d) PLA_2_s, e) SVSPs, f) C-type lectin-like proteins and g) SVMPs.

The proteomic analyses of venoms of *B*. *arietans* [17], *B*. *gabonica* [6], *B*. *nasicornis* [7], *B*. *rhinoceros* [7], and *E*. *ocellatus* [5] have been previously described. To the best of our knowledge, the complete proteomic analysis of venoms of *E*. *coloratus*, *E*. *leucogaster* and *E*. *pyramidum* has not been done, although partial analyses (i.e., identification of fractions not immunodepleted by an antivenom) of the latter two species are available [8]. The HPLC profiles of the venoms used in this study are shown in Figs 1 and S1. As expected, there are intra-species variations in the relative abundance of various fractions compared to previously published data.

In terms of toxicity, the venoms of *Bitis* spp. had similar LD_50_s, except for those of *B*. *arietans* and *B*. *nasicornis*, whose 95% CIs do not overlap (Table 1). In the case of venoms of *Echis* spp., LD_50_s were also similar, since only those of *E*. *coloratus* and *E*. *leucogaster* showed a significant difference (Table 1).

**Table 1:** Toxic activities of venoms of African *Bitis* and *Echis* species.

Venom	Lethality (LD_50_)^a^	Hemorrhage (MHD)^b^	Coagulant (MCD)^c^
*Bitis arietans*	22.0 (12.9–31.6)	0.10 ± 0.01	NAD
*Bitis gabonica*	29.4 (22.1–38.5)	0.18 ± 0.02	NAD
*Bitis nasicornis*	47.4 (33.2–67.9)	0.24 ± 0.09	NAD
*Bitis rhinoceros*	31.7 (24.9–42.0)	0.05 ± 0.01	NAD
*Echis coloratus*	22.1 (14.8–30.6)	0.31 ± 0.09	0.01 ± 0.00
*Echis leucogaster*	45.5 (33.0–62.0)	0.56 ± 0.13	0.19 ± 0.02
*Echis ocellatus*	31.2 (21.1–49.1)	0.51 ± 0.06	0.20 ± 0.05
*Echis pyramidum*	39.2 (26.4–52.2)	0.50 ± 0.08	0.13 ± 0.01

^a^ Lethality is expressed as LD_50_ (95% CI) by the i.p. route, i.e., the Median Lethal Dose, defined as the amount of venom (μg) that results in the death of 50% of the injected mice.

^b^ Hemorrhage is expressed as MHD, i.e., the Minimum Hemorrhagic Dose, defined as the amount of venom (μg) that generates 100 HaU (see Materials and Methods for details).

^c^ Coagulant activity is expressed as MCD, i.e., the Minimum Coagulant Dose, defined as the amount of venom (μg) that induces a change in absorbance of 0.01 units within 1 min. NAD means that no activity was detected. In the cases of hemorrhagic and coagulant activities, results are expressed as mean ± SD (n = 3).

*Bitis* spp. venoms had higher hemorrhagic activity than *Echis* spp. venoms (F = 21.621 _(7; 16)_, p< 0.0001; Table 1). The most hemorrhagic *Bitis* venom was *B*. *rhinoceros* (R-E-G-W Q post-hoc test p = 0.504), while the *Echis* venom having the highest hemorrhagic activity was *E*. *coloratus* (R-E-G-W Q post-hoc test p = 0.079). The hemorrhagic activity of snake venoms is due to the action of SVMPs [30, 31]. Hemorrhagic SVMPs have been isolated from the venoms of African *Bitis* spp. [32] and *Echis* spp. [33–35]. Interestingly, even though SVMPs are less abundant in venoms of *Bitis* spp. (22.9–40.9% of total venom proteins) [7] than in venoms of *Echis* spp. (60–70% of total venom proteins) [5], venoms of *Bitis* spp. are more hemorrhagic than those of *Echis* spp. (Table 1). This might be explained by the presence of procoagulant, non-hemorrhagic SVMPs in *Echis* spp. venoms [33].

Only *Echis* spp. venoms induced plasma-clotting activity *in vitro*. The venom with the highest procoagulant activity is that of *E*. *coloratus* (F = 31.925 _(3; 8)_, p< 0.0001; R-E-G-W Q post-hoc test p = 1.0; Table 1). These toxicological profiles generally agree with previous studies of African snake venoms [36–38]. Coagulant activity is due to the action of SVMPs on the coagulation cascade as prothrombin activators [39], since clotting activity of *E*. *ocellatus* venom is abrogated by SVMP inhibitors and cation chelating agents [40, 41].

In the clinical setting, coagulopathy has been reported in envenomations caused by *Echis* spp. and in some cases of envenomation caused by *Bitis* spp. in East and South Africa. In contrast, reports of envenomations by *Bitis* spp. in West Africa do not describe coagulation disorders [1, 11]. Intraspecies variation in the composition and action of venoms of *B*. *arietans* from various geographical locations has been reported [42, 43].

### Tissue damage induced by *Bitis* spp. and *Echis* spp. venoms in the immunized rabbits

Groups of four rabbits were immunized with venoms of *Bitis* spp. or *Echis* spp. The first two venom injections were administered in a water/oil (w/o) emulsion, i.e., FCA and FIA, from which venoms were slowly released, thus reducing the tissue damage caused by the venom, and enhancing the antibody response of rabbits. Assuming that the antibody response developed during the first immunizations can reduce the toxic effects of the additional venom boosters, and to minimize the inflammation caused by Freund’s adjuvants, the rest of the venom boosters were dissolved in PBS. It was therefore of interest to assess the venom-induced toxic effects in these animals.

**Fig 2:**
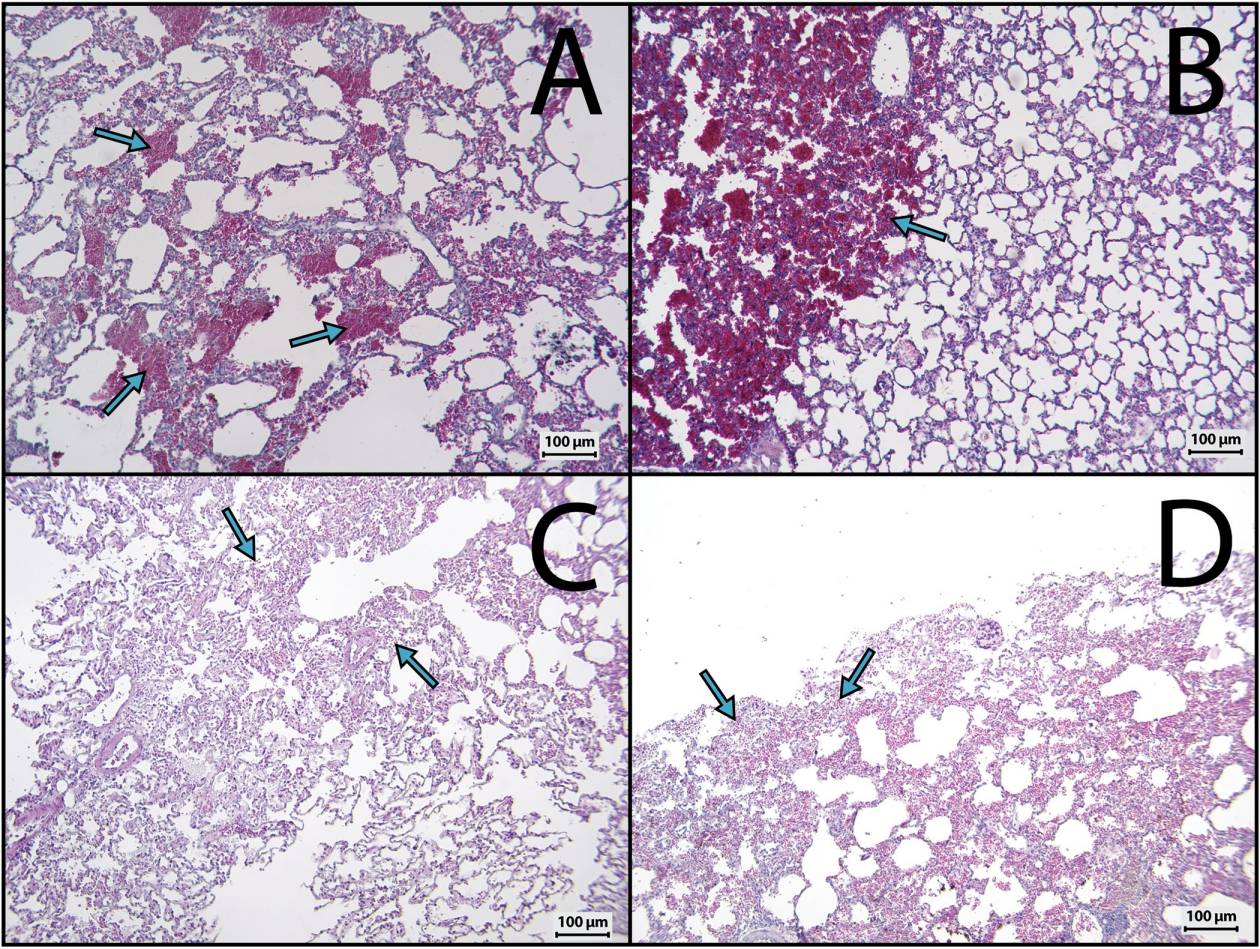
Histological alterations in lungs induced by *Bitis* spp and *Echis* spp venoms in rabbits. Immediately after euthanasia, lung samples were collected from rabbits immunized with venoms, and added to 3.7% formalin solution. After embedding the samples in paraffin, sections of 4 μm of thickness were obtained and stained with hematoxylin-eosin. Light micrographs correspond to sections obtained from rabbits immunized with the venoms of *B*. *arietans* (A), *B*. *gabonica* (B), *E*. *coloratus* (C) and *E*. *ocellatus* (D). Arrows depict areas with extravasated erythrocytes. Bar corresponds to 100 μm.

The necropsies at the end of the immunization revealed hemorrhagic lesions of varying extent at the site of venom injection and in the lungs of rabbits immunized with all *Bitis* spp. and *Echis* spp. venoms. Histologically, sections from pulmonary tissue of rabbits immunized with *Bitis* sp venoms showed more extensive hemorrhagic areas than those injected with *Echis* spp venoms (Fig 2), although extravasation in the lungs was observed for all venoms. These pathological findings agree with the higher hemorrhagic activity of *Bitis* spp venoms in mice, as described above. In contrast, no evident histological alterations were detected in skin, liver, kidneys, and heart. Hemorrhage in lungs is likely induced by PIII-SVMPs after being released from the site of injection and reached the systemic circulation. If similar damage were caused in animals used for the industrial production of snake antivenoms, these could be prevented by SVMPs inhibitors. However, this may affect epitopes and limit the neutralizing ability of antibodies, depending on the inhibitors. Another alternative to reduce toxicity would be the use of venoms detoxified by physicochemical methods, but this also has the potential risk of affecting relevant epitopes, hence reducing the ability of antibodies to neutralize the native venom [14]. Other alternatives to decrease venom toxicity during immunization would be to use low doses of venoms in more prolonged immunization schemes and to employ depot-forming adjuvants instead of PBS or saline solution to generate a stronger immune response.

**Fig 3:**
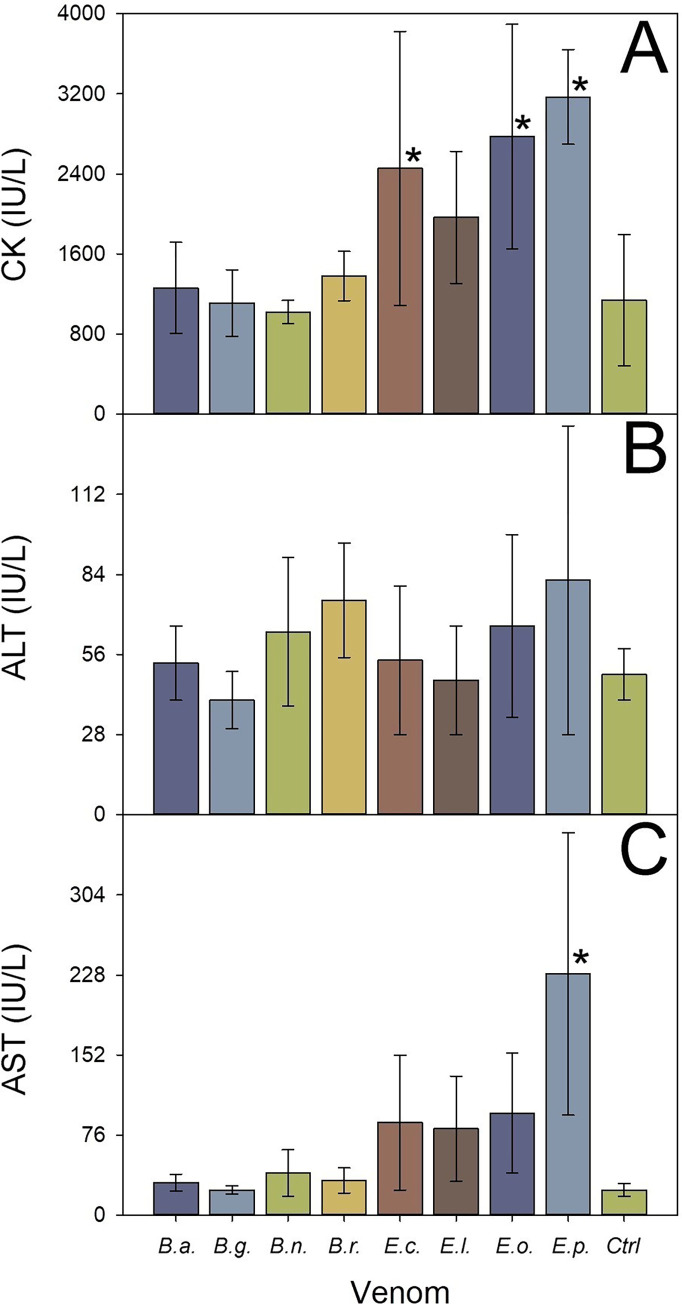
Plasma activity of enzymes in rabbits immunized with venoms. Plasma activity of A) creatine kinase (CK), B) alanine aminotransferase (ALT), and C) aspartate aminotransferase (AST) in plasma of rabbits immunized with venoms of *B*. *arietans* (B.a.), *B*. *gabonica* (B.g.), *B*. *nasicornis* (B.n.), *B*. *rhinoceros* (B.r.), *E*. *coloratus* (E.c.), *E*. *leucogaster* (E.l.), *E*. *ocellatus* (E.o.), and *E*. *pyramidum* (E.p.). Results are expressed in International Units (IU) / L and correspond to the mean ± SD (n = 4). *P < 0.05 when compared to the control group of non-immunized rabbits (Ctrl).

Muscle tissue damage during immunization was evidenced by a significant increase of the plasma CK activity, as compared to non-immunized controls (F = 4.004 _(8; 25)_, p = 0.004), in rabbits immunized with the venoms of three species of *Echis* spp. (Dunnett’s t-test: *E*. *coloratus* p = 0.048, *E*. *ocellatus* p = 0.013, *E*. *pyramidum* p = 0.011; Fig 3A). Since myotoxicity is generally caused by the action of PLA_2_s on muscle fibers, the use of PLA_2_ inhibitors during immunization would be an option to reduce the extent of muscle damage, a hypothesis that needs to be tested in large animals used in antivenom production. No differences were found between rabbits immunized either with *Bitis* spp. (F = 2.629 _(4; 15)_, p = 0.076) or *Echis* spp. (F = 0.799 _(4; 15)_, p = 0.545) venoms and the control group regarding the plasma activity of ALT (Fig 3B). Moreover, no apparent alterations on the plasma activity of AST in rabbits immunized with *Bitis* spp. venoms were observed (F = 1.211 _(4; 15)_, p = 0.347), when compared to the control group. In contrast, elevated plasma activity of AST was observed in rabbits immunized with *Echis* spp. venoms (F = 4.090 _(4; 15)_, p = 0.019), particularly in those immunized with the venom of *E*. *pyramidum* (Dunnett’s t-test p< 0.005; Fig 3C), suggesting that a degree of hepatic damage was induced by this venom during the immunization. However, histopathological analysis of liver did not reveal lesions.

At the end of the immunization, immunized rabbits showed normal values of all hematological parameters analyzed (Table A in S1 Text) [44]. Also, normal values of creatinine (F = 1.320 _(8; 27)_, p = 0.276) and urea (F = 2.034 _(8; 27)_, p = 0.08) in serum were found in all rabbits (Table B in S1 Text) [44], indicating that no significant renal injury was induced during immunization, in agreement with necropsy results. Our findings should prompt the analysis of local myonecrosis and pulmonary hemorrhage when venoms of *Bitis* spp. and *Echis* spp. are used in large animals for antivenom production. On this basis, the addition of SVMP and PLA_2_ inhibitors to the immunizing mixtures can be considered in order to limit the extent of these pathophysiological alterations [45]. However, care should be taken as to ensure that inhibitors do not alter epitopes in relevant toxins that would affect the immune response.

### Cross-reactivity and neutralization between anti-*Bitis* spp. sera

**Fig 4:**
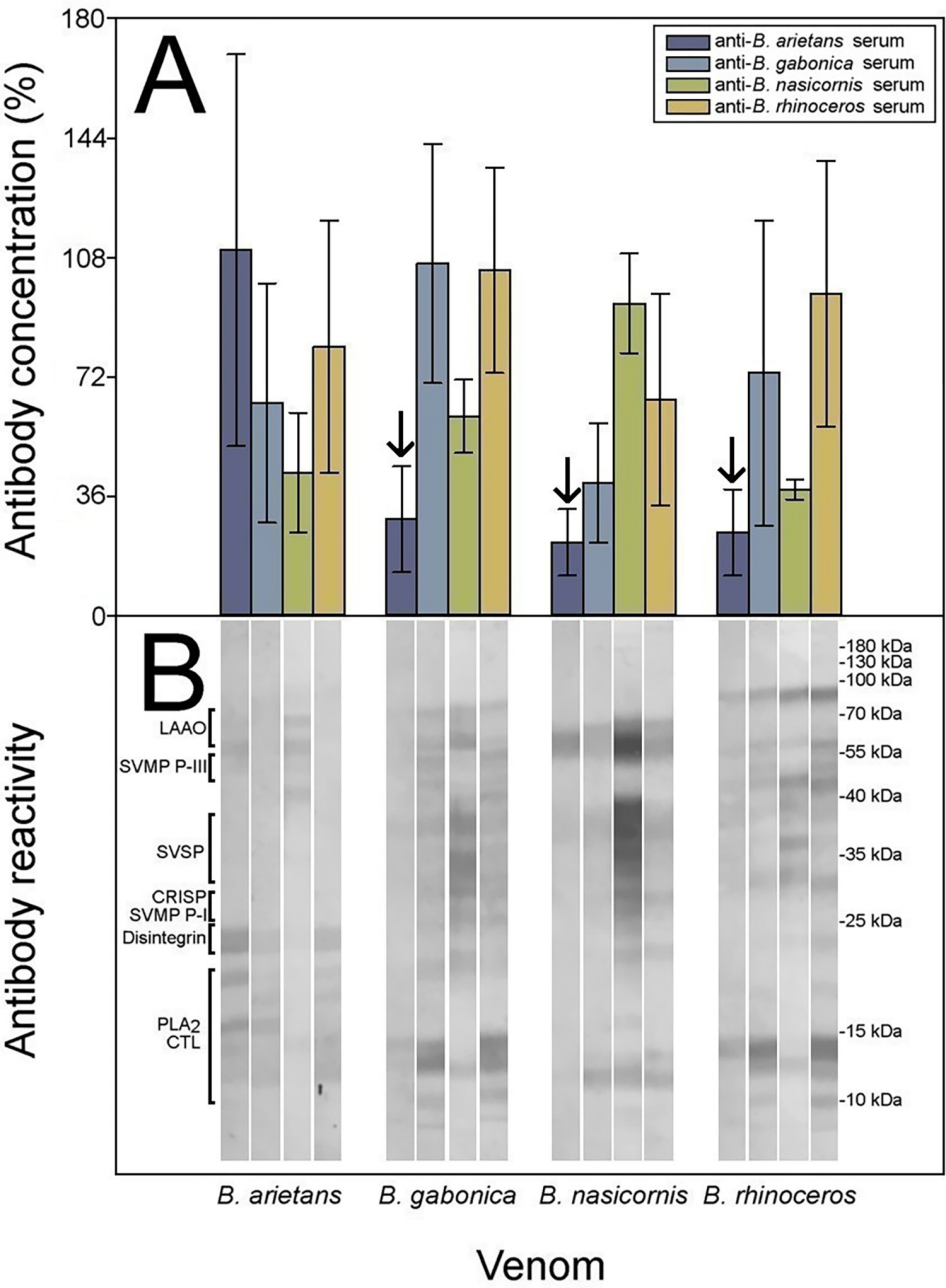
Immunochemical cross-reactivity between anti-*Bitis* spp. sera. Cross-reactivity between anti-*Bitis* spp. monospecific antisera and *Bitis* spp. venoms was determined by A) ELISA and B) Western blot. ELISA results are expressed as percentage, considering as 100% the titer of sera raised against the homologous venom of each species and correspond to the mean ± SD (n = 4). **↓** Heterologous sera with less than 33% content of specific antibodies of the corresponding homologous serum. LAAO: L-amino acid oxidases, SVMP: Zn^2+^-dependent snake venom metalloproteinases, SVSP: snake venom serine proteinases, CRISP: cysteine-rich secretory proteins, PLA_2_: phospholipases A_2_, CTL: C-type lectin-like proteins.

Our experimental protocol, which combines antibody titers by ELISA, Western blot, and neutralization of toxic activities, allowed the assessment of the intrageneric cross-reactivity between monospecific rabbit sera against homologous and heterologous venoms. Cross-reactivity was observed between the four anti-*Bitis* antisera against the four *Bitis* spp. venoms, revealing a clear antigenic similarity of the venoms, although there were quantitative differences between them (Fig 4A and 4B).

As expected, the strongest antibody responses were observed against the homologous venoms. The monospecific anti-*B*. *gabonica* (F = 8.485 _(3; 12)_, p = 0.003), and anti-*B*. *nasicornis* (F = 9.402 _(3; 12)_, p = 0.002) antisera showed a strong cross-reactivity by ELISA against heterologous venoms, while the anti-*B*. *rhinoceros* antiserum showed the strongest cross-reactivity (F = 4.434 _(3; 12)_, p = 0.026; Fig 4A). In contrast, the anti-*B*. *arietans* serum showed the lowest extent of cross-reactivity (F = 1.982 _(3; 12)_, p = 0.170). This antiserum presented reactivities lower than the arbitrarily pre-established limit of 33%, selected as our threshold value, against heterologous venoms (R-E-G-W Q post-hoc test p = 0.139), and therefore was not assessed in the *in vivo* neutralization assays to avoid the unnecessary use of mice.

Cross-reactivity was also evident in Western blot. Presumptive identification of the immunoreactive protein bands, based on the molecular masses, is presented in Fig 4B, according to Mackessy [46]. In agreement with ELISA results, stronger reactions were observed by Western blot when antisera were tested against homologous venoms, and a weak reaction was seen in the case of *B*. *arietans* antiserum when confronted with the other venoms (Fig 4B).

**Fig 5:**
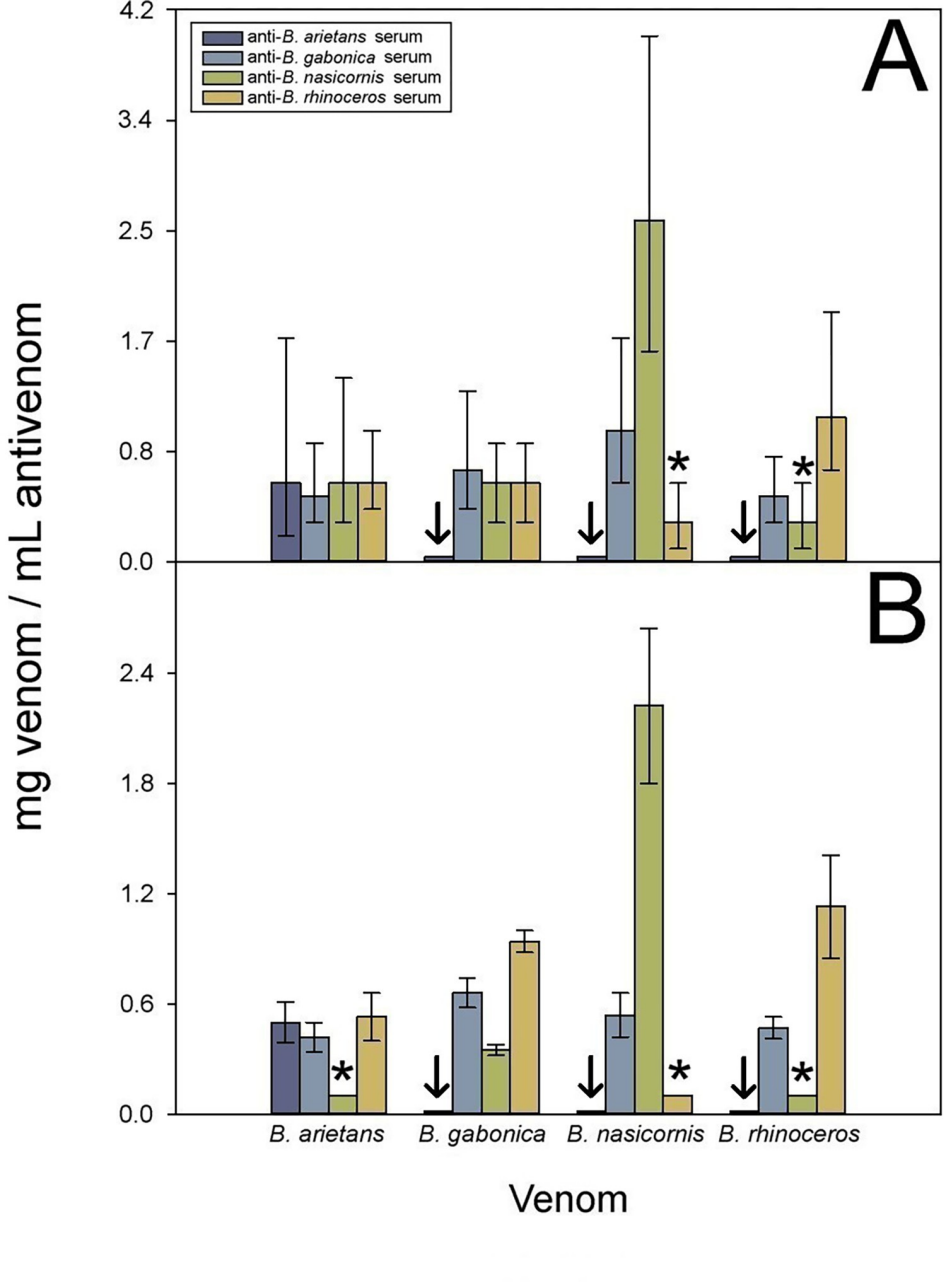
Cross-neutralization of toxic activities of *Bitis* spp. venoms by monospecific antisera. Cross-neutralization of A) lethal and B) hemorrhagic activities of *Bitis* spp. venoms. Only the antisera that gave higher than 33% cross-reactivity by ELISA were tested in these assays. Neutralization is expressed as ED_50_, i.e., mg venom neutralized per mL antivenom. In the case of lethality, error bars represent the 95% confidence intervals, while in the case of hemorrhage, error bars represent SD (n = 4). *Non-overlapping values with the anti-lethal 95% CI of the homologous serum, or no detectable ability to neutralize the hemorrhagic activity. ↓ heterologous sera that were no tested as they have less than 33% content of specific antibodies of the corresponding homologous serum.

When cross-neutralization of toxic effects was analyzed, it was found that anti-*B*. *gabonica*, anti-*B*. *nasicornis*, and anti-*B*. *rhinoceros* neutralized lethality of the four venoms, with varying values of ED_50_s, but with a high extent of overlap in the 95% CI (Fig 5A). Exceptions were the anti-*B*. *rhinoceros* antiserum against the venom of *B*. *nasicornis*, and the anti-*B*. *nasicornis* antiserum against the venom of *B*. *rhinoceros*, whose 95% CIs did not overlap with those of the corresponding homologous systems.

Regarding neutralization of hemorrhagic activity, the antisera neutralized the homologous and most of the heterologous venoms assessed, with different ED_50_s (F _*B*. *arietans*_ = 5.269 _(3; 8)_, p = 0.027; F _*B*. *gabonica*_ = 103.724 _(3; 8)_, p< 0.0001; F _*B*. *nasicornis*_ = 56.695 _(3; 8)_, p = 0.0001; F _*B*. *rhinoceros*_ = 23.020 _(3; 8)_, p = 0.0001). This result highlights structural conservation of SVMPs, which is expected in snakes of close phylogenetic relationships. When compared to the corresponding homologous antisera, the anti-*B*. *gabonica* antiserum showed lower ability to neutralize the hemorrhagic activity of the venoms of *B*. *nasicornis* (*t* = 6.121_(4)_, p = 0.004) and *B*. *rhinoceros* (*t* = -4.828_(4)_, p = 0.008). A similar case was that of anti-*B*. *nasicornis* antiserum against the venom of *B*. *gabonica* (*t* = 6.679_(4)_, p = 0.003). In contrast, no neutralization was detected in the case of the anti-*B*. *nasicornis* antiserum against venoms of *B*. *arietans* (*t* = 8.161_(4)_, p = 0.001) and *B*. *rhinoceros* (*t* = 8.161_(4)_, p = 0.001) venoms; and anti-*B*. *rhinoceros* serum failed to neutralize the activity of *B*. *nasicornis* (*t* = 5.147_(4)_, p = 0.007; Fig 5B). In these cases, the difference/similarity balance between SVMPs suggest that several venoms should be used as immunogens to generate an antivenom with the ability to neutralize hemorrhagic activity of venoms of several *Bitis* species. All mean values and SD used for Figs 4 and 5 are included in the Tables C, D and E in S1 Text.

Taken together, the data on ELISA and neutralization of lethality and hemorrhage indicate that the anti-*Bitis* antisera can be arranged from highest to lowest cross-reactivity as follows: anti-*B*. *gabonica*, anti-*B*. *rhinoceros*, anti-*B*. *nasicornis*, and anti-*B*. *arietans*.

### Cross-reactivity and neutralization between anti-*Echis* spp. sera

**Fig 6:**
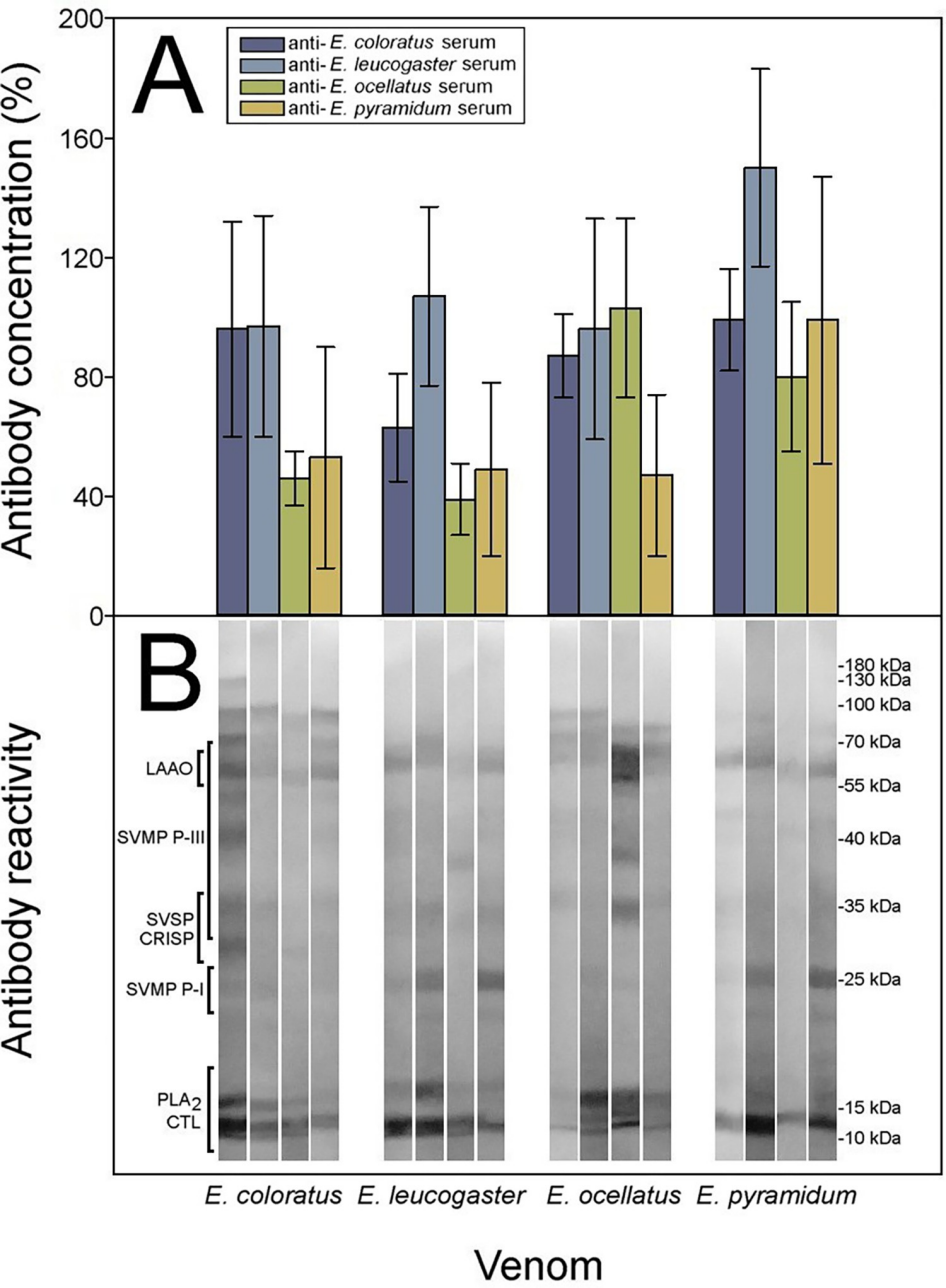
Immunochemical cross-reactivity between anti-*Echis* spp. sera. Cross-reactivity between anti-*Echis* spp. monospecific antisera and *Echis* spp. venoms determined by A) ELISA and B) Western blot. ELISA results are expressed as a percentage, considering as 100% the titer of serum raised against the homologous venom of each species and correspond to the mean ± SD (n = 4). All heterologous antisera have > 33% content of specific antibodies of the corresponding homologous antiserum. LAAO: L-amino acid oxidases, SVMP: Zn^2+^-dependent snake venom metalloproteinases, SVSP: snake venom serine proteinases, CRISP: cysteine-rich secretory proteins, PLA_2_: phospholipases A_2_, CTL: C-type lectin-like proteins.

As in the case of *Bitis* spp. venoms, an extensive cross-reactivity was observed between anti-*Echis* spp. antisera when confronted with the four *Echis spp*. venoms, as judged both by ELISA and Western blot, revealing a close antigenic similarity between these venoms (Fig 6A and 6B). A range of immunoreactive bands of various molecular masses, corresponding to different venom components, was observed in Western blot analysis (Fig 6B). As with *Bitis* spp. venoms, the number and intensity of the immunoreactive bands were higher in homologous venom-antiserum systems, as compared to heterologous systems. Since ELISA results showed cross-reactivities higher than 33% for all antisera against heterologous venoms when compared to homologous antisera, especially with cross-reactivities shown by anti-*E*. *leucogaster* (F = 6.461 _(3; 12)_, p = 0.008) antiserum, all venoms and antisera were then subjected to experiments of neutralization of toxic activities.

**Fig 7:**
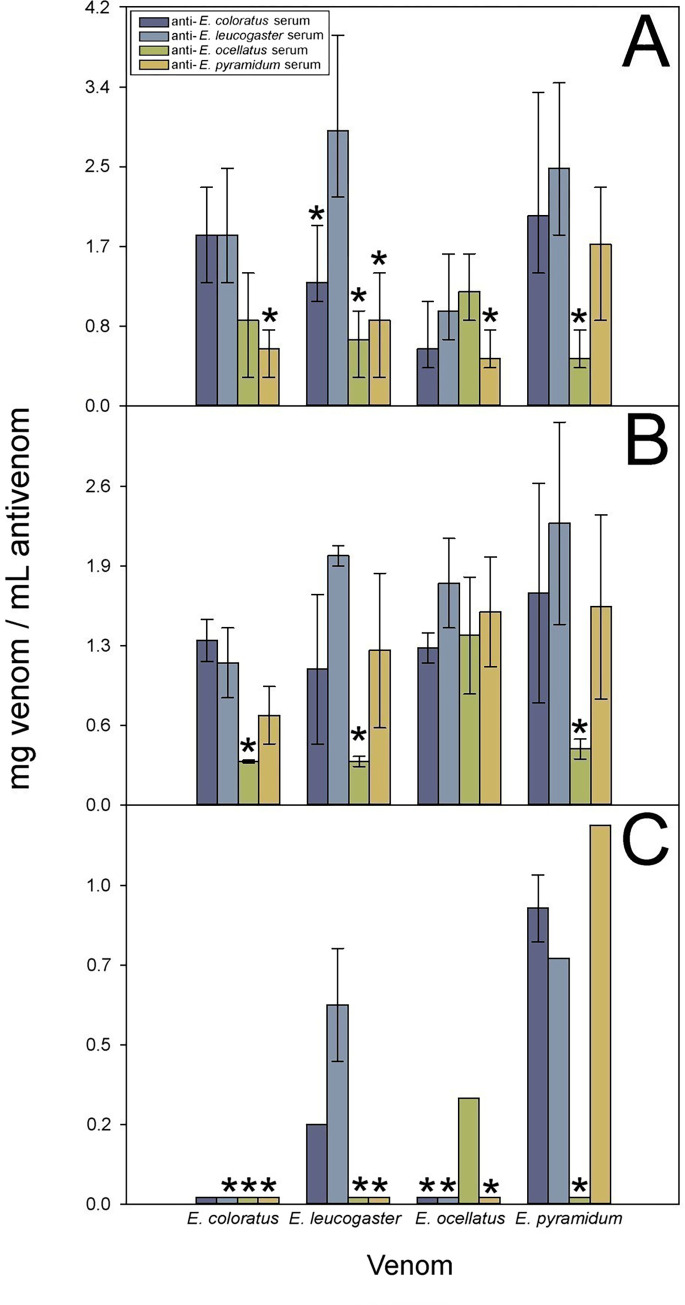
Cross-neutralization of toxic activities of *Echis* spp. venoms. Cross-neutralization of A) lethal, B) hemorrhagic, and C) coagulant activities of the *Echis* spp. venoms. Neutralization is expressed as ED_50_ (lethality and hemorrhage) and ED (coagulant effect), i.e., mg venom neutralized per mL antivenom. In the case of lethality, error bars represent the 95% confidence intervals, while in the case of hemorrhage and coagulation, error bars represent SD (n = 4). * Non-overlapping values with the anti-lethal 95% CI of the homologous serum, or no detectable or poor ability to neutralize hemorrhagic or coagulant activities.

Regarding the neutralization of lethality, all antisera were capable of neutralizing homologous and heterologous venoms, albeit with significant variations in the values of ED_50_ (Fig 7A). This agrees with a previous study in which a high extent of cross reactivity and neutralization of a monospecific anti-*E*. *ocellatus* serum against the venoms of *E*. *coloratus* and *E*. *pyramidum* was demonstrated [38].

The 95% CI did not overlap with those of the corresponding homologous systems in the case of the anti-*E*. *pyramidum* antiserum against the venoms of *E*. *coloratus*, *E*. *leucogaster* and *E*. *ocellatus*, the anti-*E*. *ocellatus* antiserum against the venoms of *E*. *leucogaster* and *E*. *pyramidum*, and the anti-*E*. *coloratus* antiserum against the venom of *E*. *leucogaster*.

A similar trend was observed in the neutralization of hemorrhagic activity, where an extensive cross-neutralization was observed, although with variable ED_50_s (F _*E*. *coloratus*_ = 14.356 _(3; 8)_, p = 0.001; F _*E*. *leucogaster*_ = 7.349 _(3; 8)_, p< 0.011; F _*E*. *ocellatus*_ = 1.104 _(3; 8)_, p = 0.402; F _*E*. *pyramidum*_ = 3.462 _(3; 8)_, p = 0.071). An exception was the anti-*E*. *ocellatus* antiserum which showed low efficacy against the venoms of *E*. *coloratus* (*t* = 0.320_(4)_, p = 0.765), *E*. *leucogaster* (*t* = -1.239_(4)_, p = 0.283), and *E*. *pyramidum* (*t* = -0.516_(4)_, p = 0.633; Fig 7B).

The coagulant activity of venom of *E*. *coloratus* was not neutralized by any of the antisera, including the homologous one (Fig 7C). Moreover, the coagulant activity of the venom of *E*. *leucogaster* was neutralized by the homologous antisera and the anti-*E*. *coloratus* antiserum (*t* = 3.600_(4)_, p = 0.023; Fig 7C). The coagulant activity of the venom of *E*. *ocellatus* was neutralized only by the homologous antiserum (Fig 7C). Finally, the coagulant activity of *E*. *pyramidum* was neutralized by the anti-*E*. *coloratus* (*t* = 4.750_(4)_, p = 0.009) and anti-*E*. *leucogaster* (*t* = 4.000_(4)_, p = 0.016) sera and the homologous antiserum. These findings underscore a complex pattern of antigenic variation in the coagulant toxins present in these venoms. Taken together, our data indicate that the anti-*Echis* antisera can be ordered from most to least cross-reactive as follows: anti-*E*. *leucogaster*, anti-*E*. *coloratus*, anti-*E*. *pyramidum*, and anti-*E*. *ocellatus*. All mean values and SD used for Figs 6 and 7 are included in the Tables F, G, H and I in S1 Text.

The extensive cross-reactivity of the monospecific antisera generated in this work agrees with previous studies where antivenoms generated by using different mixtures of venoms neutralized lethal, hemorrhagic, and coagulant effects of *Bitis* spp. and *Echis* spp. venoms [29, 36, 47, 48]. According to our findings, however, the venom of *B*. *arietans* generated a relatively limited immune response in rabbits when tested against venoms of the other three *Bitis* species. Likewise, our results underscore the limited extent of cross-neutralization of coagulant activity of *Echis* spp. venoms by heterologous antisera, in agreement with previous findings showing a narrow taxonomic range of efficacy of these antisera to neutralize *in vitro* coagulant effect of *Echis* spp. venoms [49].

## Conclusions

Our results underscored the toxic effects induced by venoms in immunized rabbits, especially regarding myonecrosis and systemic hemorrhage. This raises the possibility of introducing the use of inhibitors of venom enzymes, i.e., SVMPs and PLA_2_s, which would reduce toxicity, but ensuring that this does not affect immunogenicity in antivenom production, as previously shown in the case of a chelating agent [45]. The main goal of this study was to infer the intrageneric antigenic relatedness between venoms of species of African *Bitis* spp and *Echis* spp based on the analysis of the cross-reactivity of monospecific sera generated in rabbits. The protocol can be used for inferring antigenic similarities of venoms of clinically relevant snake species for other venoms in sub-Saharan Africa and other regions as well. This information can be complemented by other approaches to determine antigenic similarities between venoms, such as antivenomics [8, 48].

Our observations demonstrate a remarkable cross-reactivity between monospecific antisera and the most important venoms of *Bitis* and *Echis* species in sub-Saharan Africa. Cross-reactivity between monospecific anti-*Bitis* spp. antisera suggests antigenic similarities between lethal and hemorrhagic toxins of venoms of *B*. *arietans*, *B*. *gabonica*, *B*. *nasicornis*, and *B*. *rhinoceros*. In a similar way, but with some exceptions, cross-reactivity between monospecific anti-*Echis* spp antisera suggests antigenic similarity between lethal and hemorrhagic toxins of venoms of *E*. *coloratus*, *E*. *leucogaster*, *E*. *ocellatus*, and *E*. *pyramidum*, but not in their procoagulant toxins. As a broad conclusion, the highest immune coverage within each genus was observed with the venoms of *B*. *gabonica*, *B*. *rhinoceros*, and *E*. *leucogaster*. However, it is likely that the production of antivenoms with the widest neutralizing scope will require the use of venoms from additional species of *Bitis* and *Echis*, a hypothesis that will be explored in future studies.

The rabbit immunization models are pragmatic alternatives to generate preliminary results that allow the formulation of rational hypotheses regarding the design of the immunization strategy to produce broad neutralizing antivenoms. However, as immunogenicity depends on the nature of the immune system of the animal selected as source of immunoglobulins, the conclusions of our experiments in rabbits cannot be directly extrapolated to the species used in industrial production of antivenoms. Experimental evaluation of these hypotheses in horses or sheep is required.

## Supporting information

S1 Fig RP-HPLC profiles of the venoms of *Bitis* sp and *Echis* sp included in this study (see Materials and Methods for details). (PDF)

S1 Text Table A: Hematological parameters of rabbits at the end of the immunization with venoms of African *Bitis* or *Echis* species. Table B: Plasma biochemical parameters of rabbits at the end of the immunization with venoms of African *Bitis* or *Echis* species. Table C: Cross-reactivity by ELISA between anti-*Bitis* spp. sera. Table D: Cross-neutralization of lethality of venoms of African *Bitis* species. Table E: Cross-neutralization of hemorrhagic activity of venoms of African *Bitis* species. Table F: Cross-reactivity by ELISA between anti-*Echis* spp. sera. Table G: Cross-neutralization of lethality of venoms of African *Echis* species. Table H: Cross-neutralization of hemorrhagic activity of venoms of African *Echis* species. Table I: Cross-neutralization of coagulant activity of venoms of African *Echis* species.
(DOCX)
