# Supplementary material for: Intrageneric cross-reactivity of monospecific rabbit antisera against venoms of the medically most important Bitis spp. and Echis spp. African snakes
Source: PLoS Negl Trop Dis. 2022 Aug 12;16(8):e0010643. doi: 10.1371/journal.pntd.0010643 (PMC9374258; doi:10.1371/journal.pntd.0010643)

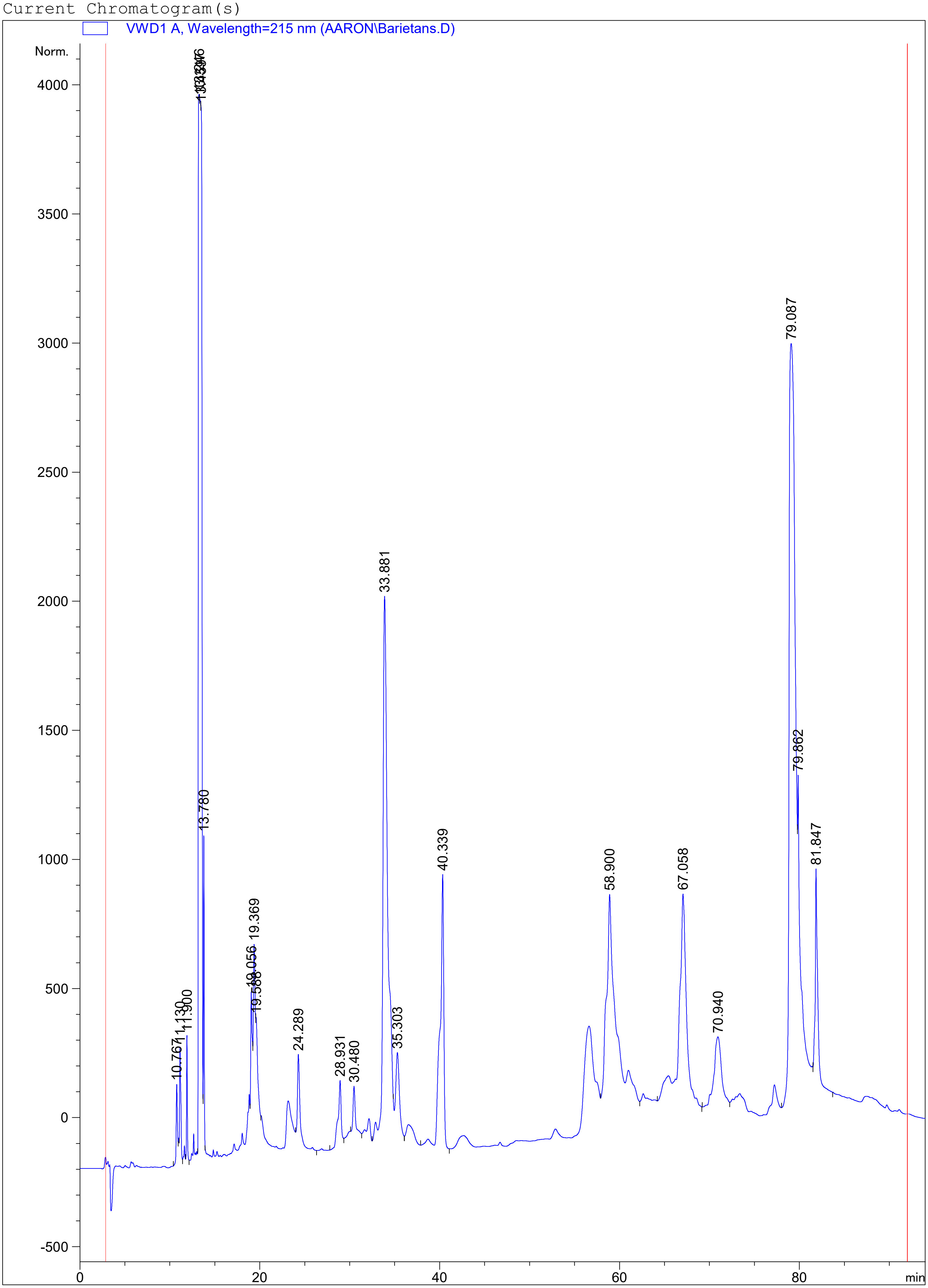
Current Chromatogram(s)
VWD1 A, Wavelength=215 nm (AARON\Barietans.D)
Norm.
4000
3500
3000
2500
2000
1500
1000
500
0
-500
0
20
40
60
80
min
10.767
11.130
11.900
13.780
19.056
19.369
19.588
24.289
28.931
30.480
33.881
35.303
40.339
58.900
67.058
70.940
79.087
79.862
81.847

Current Chromatogram(s)

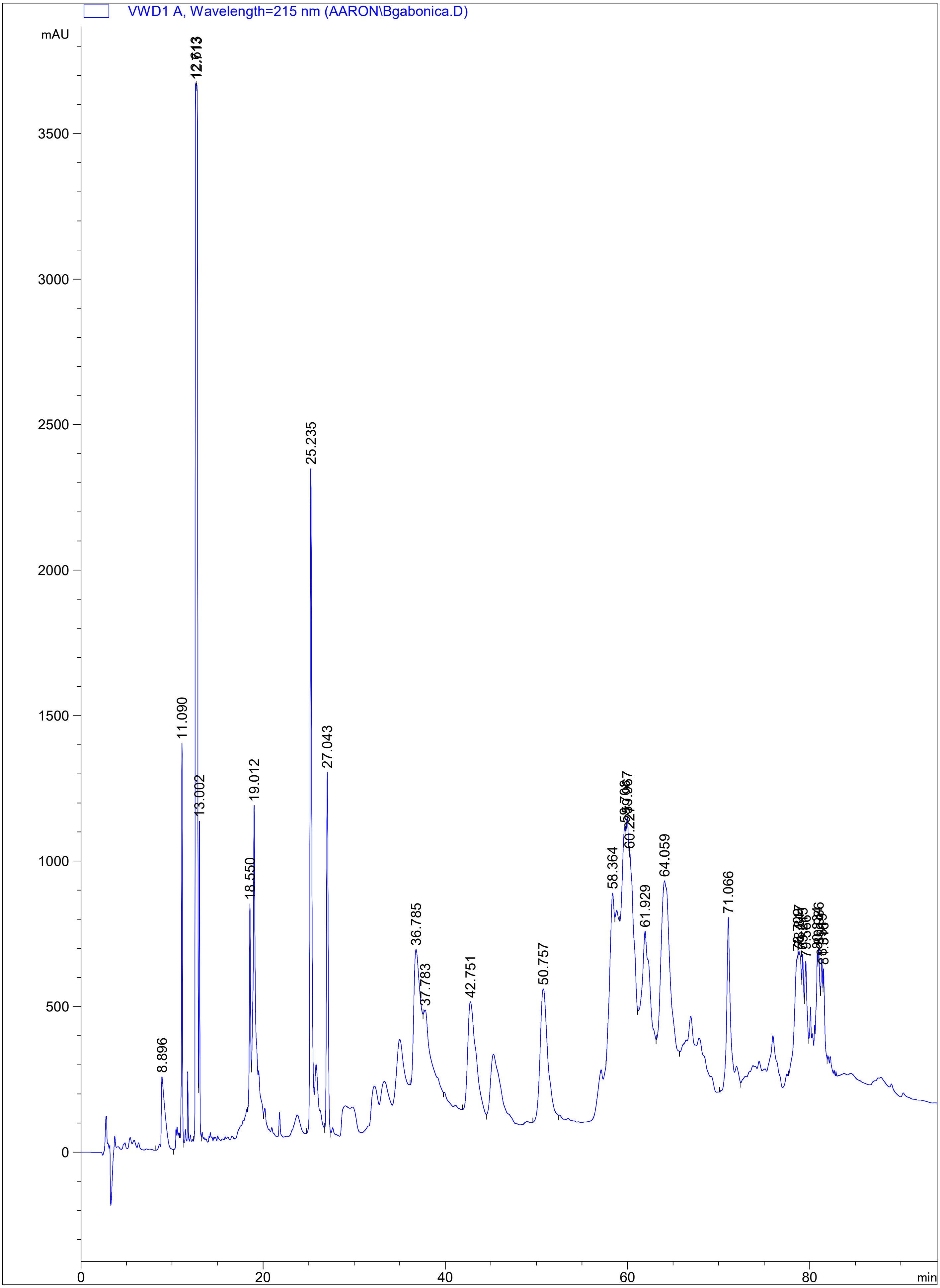

Current Chromatogram(s)

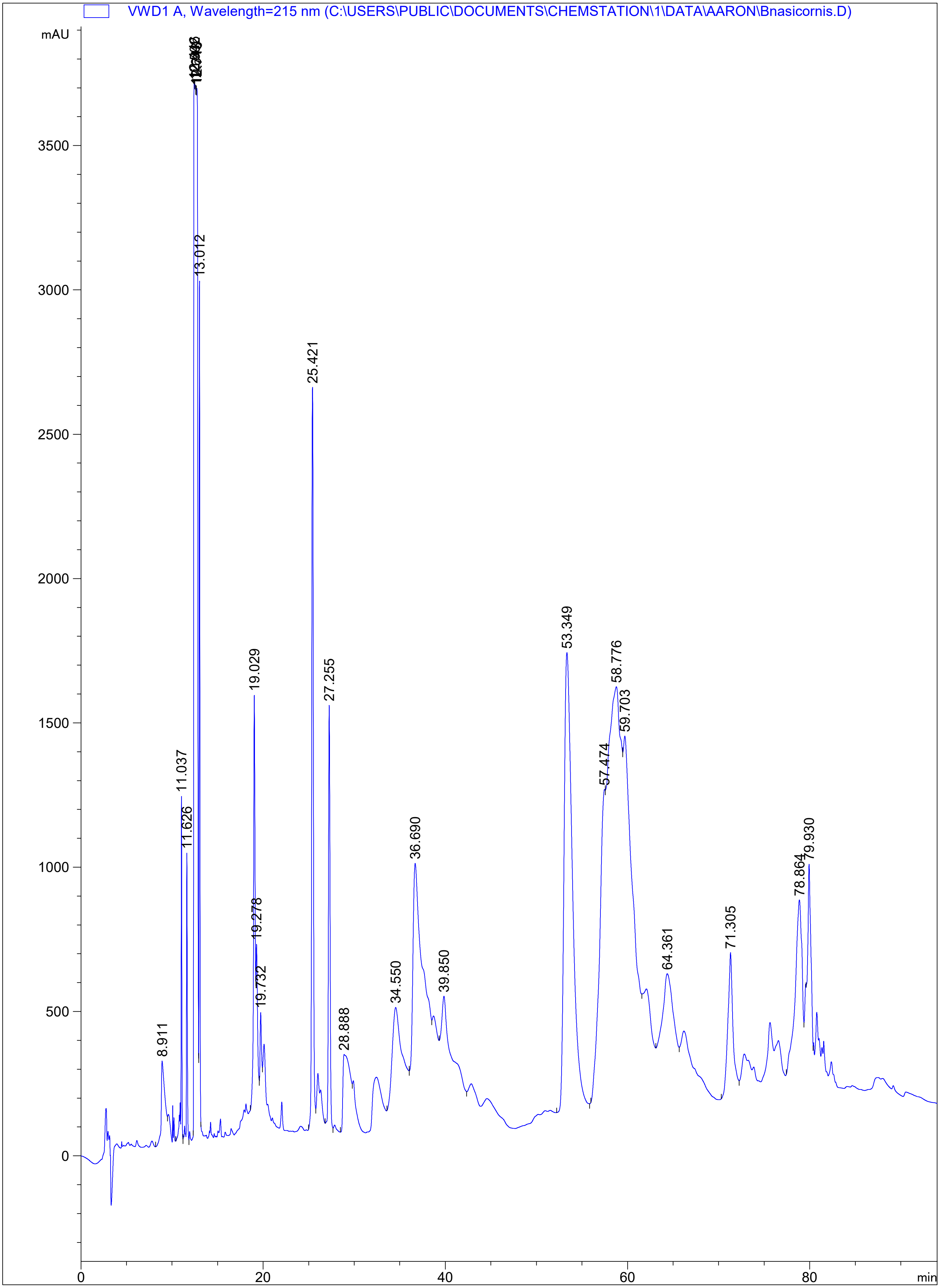

Current Chromatogram(s)

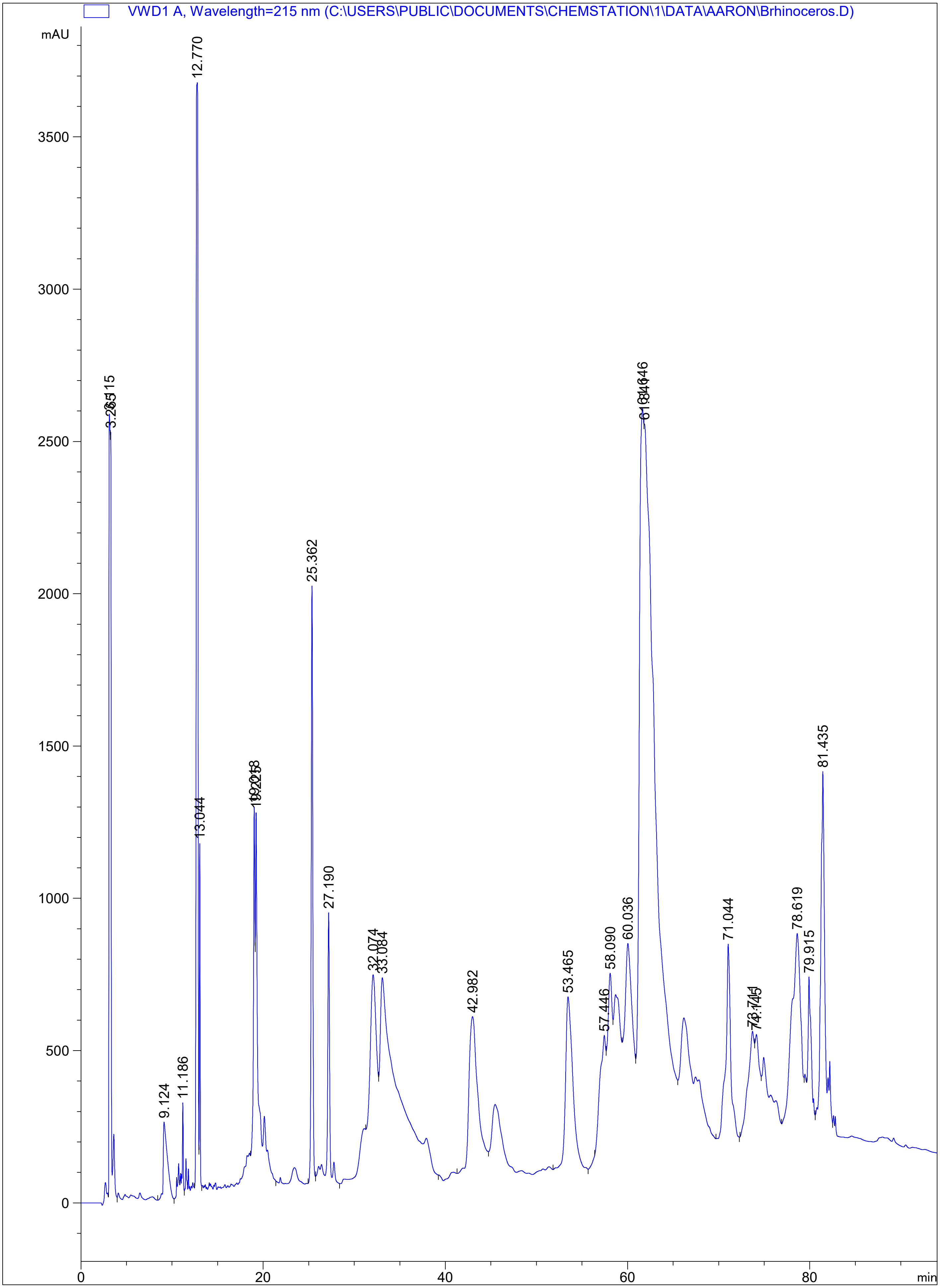

Current Chromatogram(s)

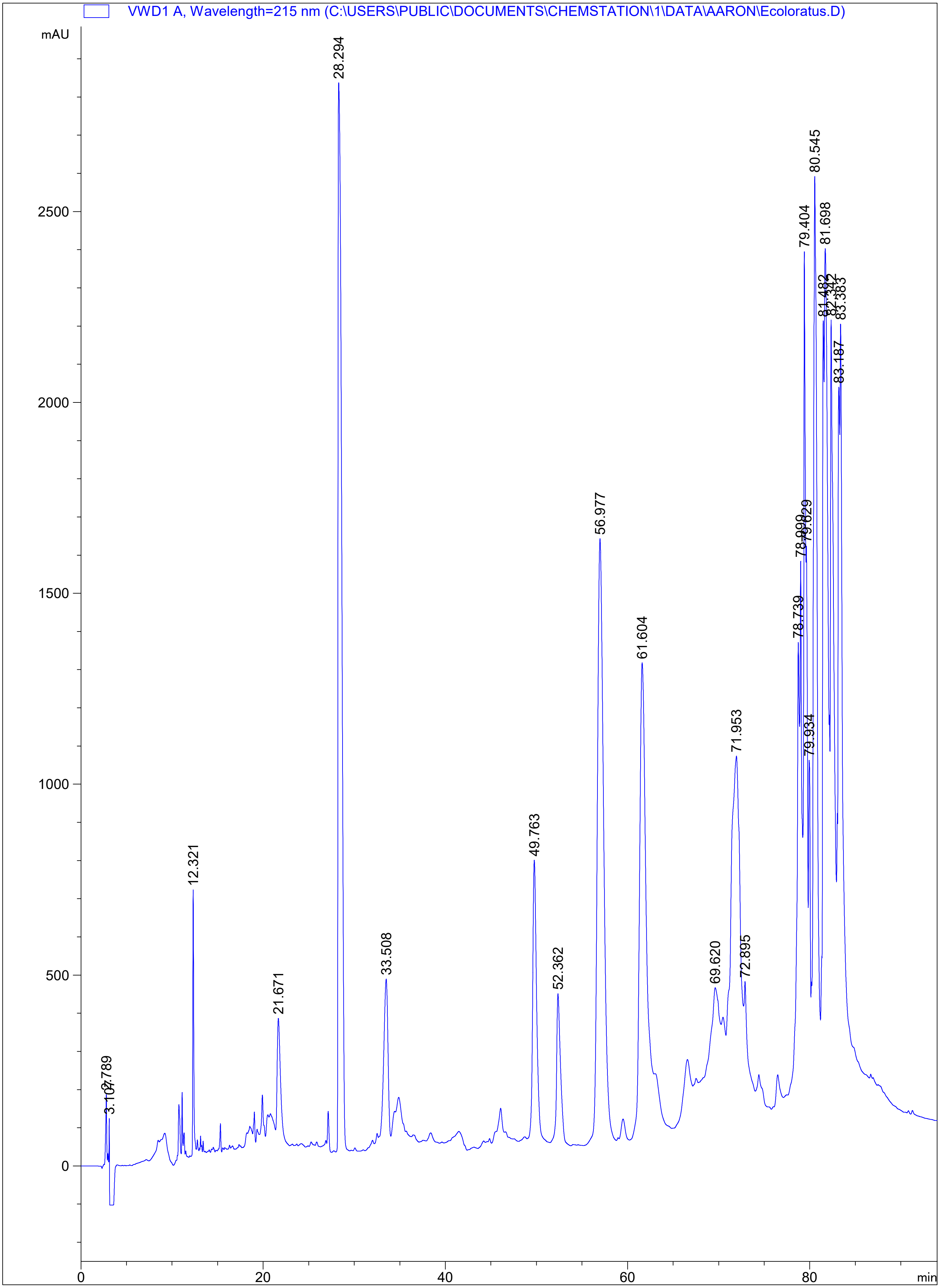

Current Chromatogram(s)

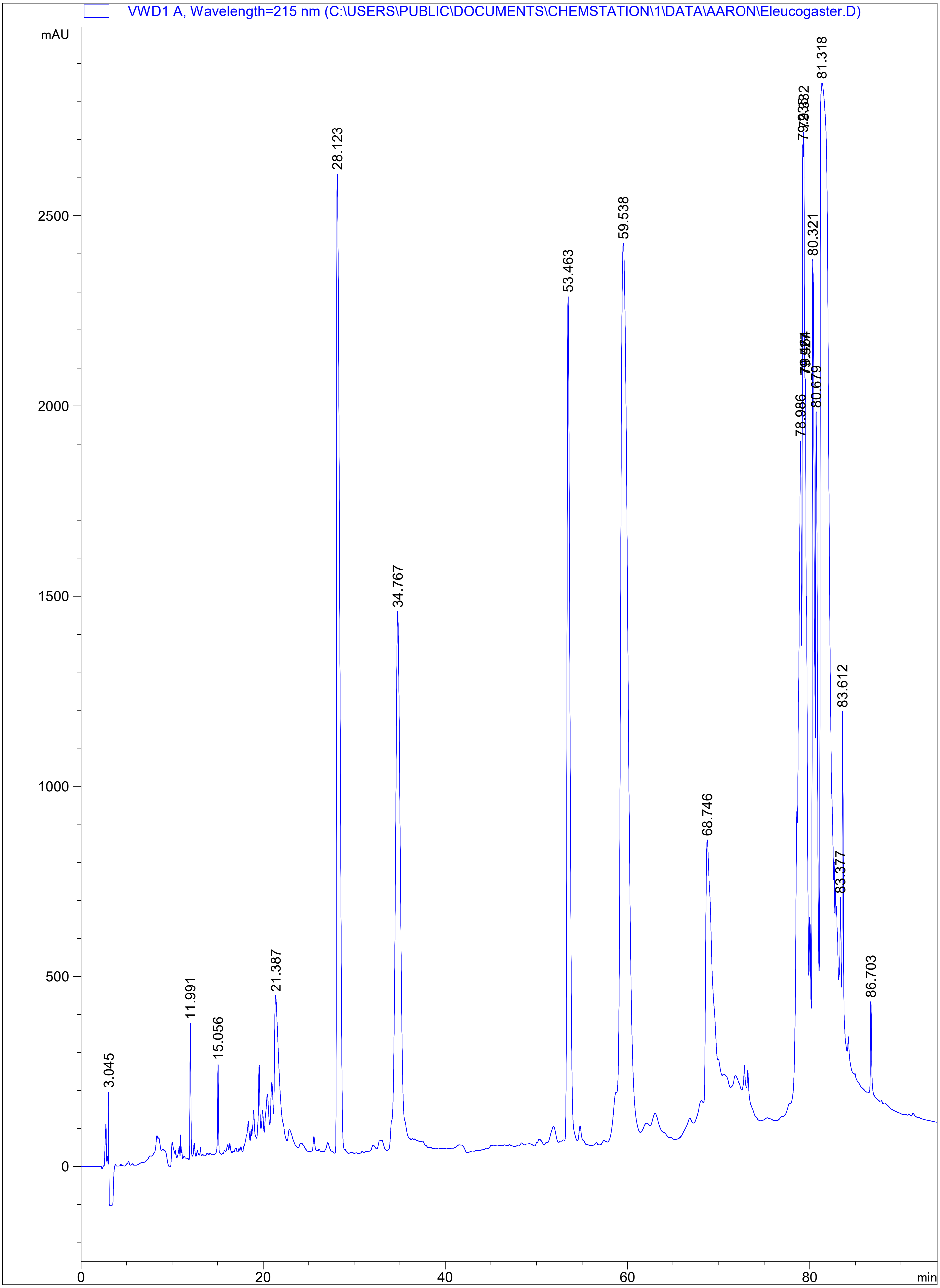

Current Chromatogram(s)

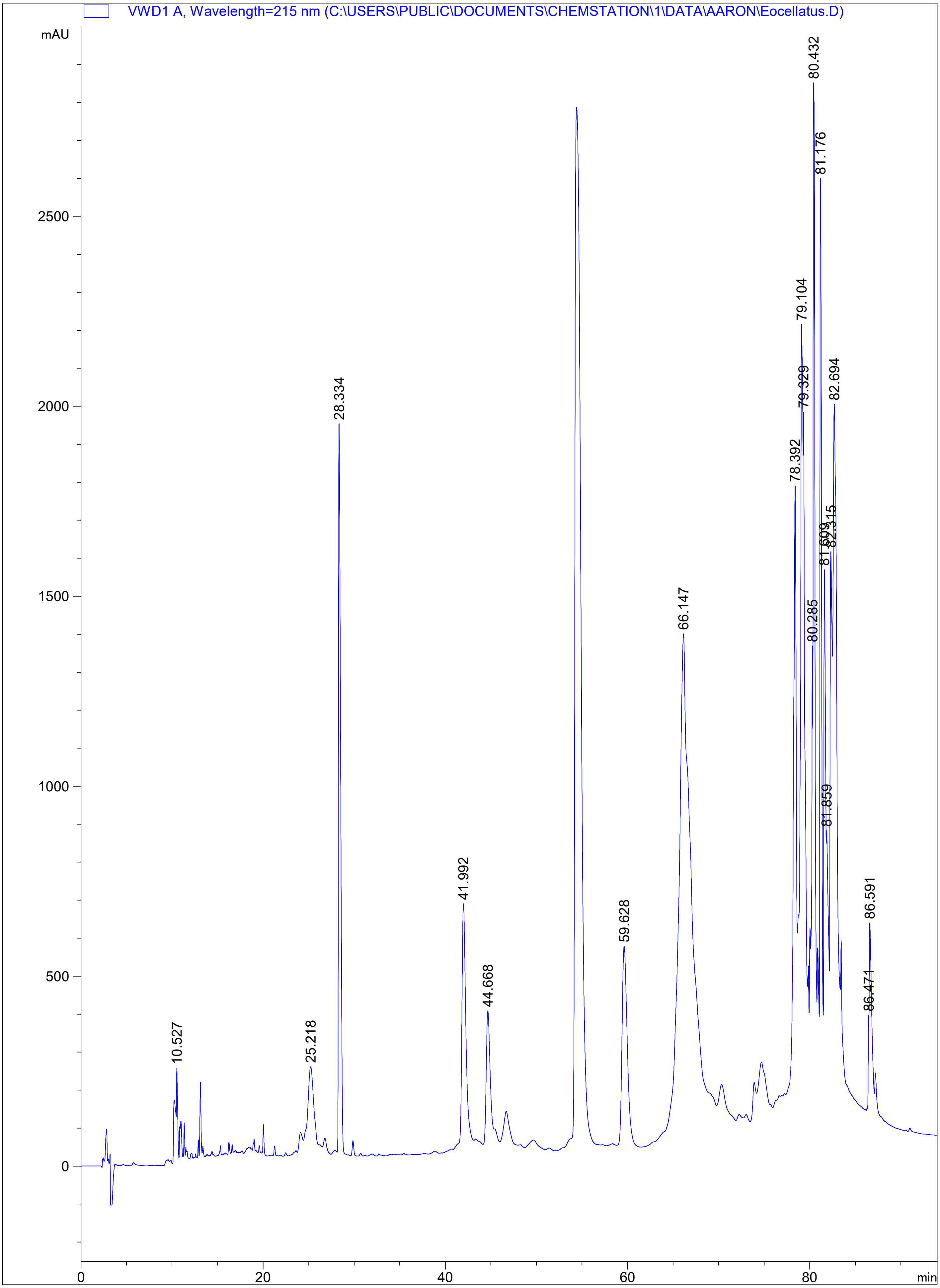

Current Chromatogram(s)

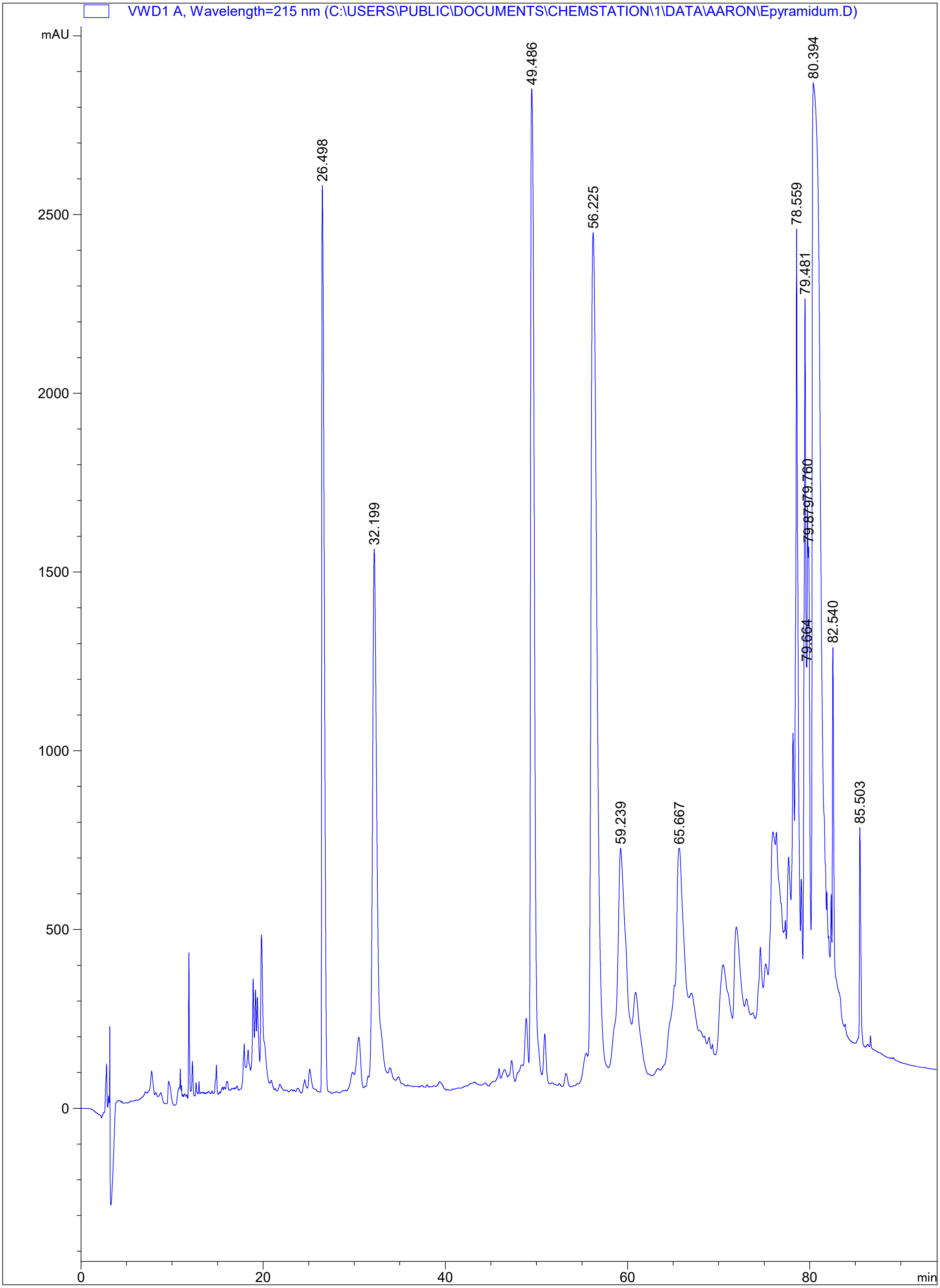

Supplement: S1 Fig — (PDF) [file pntd.0010643.s001.pdf]
