# Supplementary material for: Intrageneric cross-reactivity of monospecific rabbit antisera against venoms of the medically most important Bitis spp. and Echis spp. African snakes
Source: PLoS Negl Trop Dis. 2022 Aug 12;16(8):e0010643. doi: 10.1371/journal.pntd.0010643 (PMC9374258; doi:10.1371/journal.pntd.0010643)
Supplement: S1 Text — Table A: Hematological parameters of rabbits at the end of the immunization with venoms of African Bitis or Echis species. Table B: Plasma biochemical parameters of rabbits at the end of the immunization with venoms of African Bitis or Echis species. Table C: Cross-reactivity by ELISA between anti-Bitis spp. sera. Table D: Cross-neutralization of lethality of venoms of African Bitis species. Table E: Cross-neutralization of hemorrhagic activity of venoms of African Bitis species. Table F: Cross-reactivity by ELISA between anti-Echis spp. sera. Table G: Cross-neutralization of lethality of venoms of African Echis species. Table H: Cross-neutralization of hemorrhagic activity of venoms of African Echis species. Table I: Cross-neutralization of coagulant activity of venoms of African Echis species. (DOCX) [file pntd.0010643.s002.docx]

**S1 Text**

Table A: Hematological parameters of rabbits at the end of the immunization with venoms of African *Bitis* or *Echis* species

| Rabbit  Group* | Hematologic parameters | | | | | | |
| --- | --- | --- | --- | --- | --- | --- | --- |
|  | Ht  (%) | Hb  (g/L) | RBC  (10^6^/μL) | WBC  (10^3^/μL) | LR  (%) | GR  (%) | PLT  (10^3^/μL) |
| anti-Baa | 35 ± 2 | 12.2 ± 0.4 | 5.5 ± 0.3 | 8.6 ± 1.9 | 30 ± 6 | 60 ± 6 | 403 ± 69 |
| anti-Bg | 40 ± 2 | 14.0 ±0.6 | 6.7 ± 0.3 | 10.0 ± 2.3 | 41 ± 6 | 49 ± 5 | 417 ± 114 |
| anti-Bn | 33 ± 2 | 11.6 ± 0.2 | 5.2 ± 0.2 | 8.8 ± 2.2 | 35 ± 13 | 55 ± 12 | 348 ± 40 |
| anti-Br | 34 ± 3 | 12.0 ± 1.1 | 5.5 ± 0.3 | 9.0 ± 0.3 | 40 ± 10 | 53 ± 10 | 412 ± 109 |
| anti-Ec | 42 ± 7 | 13.8 ± 2.1 | 6.4 ± 1.1 | 8.1 ± 0.6 | 46 ± 7 | 45 ± 8 | 414 ± 108 |
| anti-El | 39 ± 6 | 13.0 ± 1.8 | 5.8 ± 0.9 | 10.7 ± 1.8 | 37 ± 11 | 53 ± 11 | 354 ± 166 |
| anti-Eo | 35 ± 4 | 11.5 ± 0.9 | 5.1 ± 0.3 | 8.1 ± 0.6 | 38 ± 2 | 52 ± 3 | 256 ± 55 |
| anti-Ep | 41 ± 4 | 12.8 ± 1.5 | 6.1 ± 0.7 | 7.5 ± 1.1 | 46 ± 6 | 44 ± 6 | 316 ± 49 |
| Control** | 37 ± 7 | 12.0 ± 2.4 | 6.0 ± 1.5 | 6.1 ± 1.8 | 36 ± 7 | 54 ± 6 | 453 ± 136 |
| Reference*** | 27-47 | 8.9-15.5 | 3.7-7.5 | 5.2-16.5 | --- | --- | 112-795 |

Ht: Hematocrit; Hb: Hemoglobin; RBC: Erythrocyte count; WBC: Leukocyte count; LR: Relative lymphocytes count; GR: Relative granulocytes count; PLT: Platelet count.

*Baa: *Bitis arietans arietans*; Bg: *B. gabonica*; Bn: *B. nasicornis*; Br: *B. rhinoceros*; Ec: *Echis c*oloratus; El: *E. leucogaster*; Eo: *E. ocellatus*; Ep: *E. pyramidum*

**Control refers to a group of non-immunized rabbits.

***Reference range for New Zealand rabbits [1].

Table B: Plasma biochemical parameters of rabbits at the end of the immunization with venoms of African *Bitis* or *Echis* species

| Rabbit  Group* | Biochemical parameter | | | | | | |
| --- | --- | --- | --- | --- | --- | --- | --- |
|  | Urea  (mmol/L) | Creatinine  (μmol/L) | ALT  (U/L) | AST  (U/L) | ALP  (U/L) | Albumin  (g/L) | CK  (U/L) |
| anti-Baa | 6.3 ± 0.5 | 84 ± 12 | 53 ± 13 | 31 ± 8 | 112 ± 46 | 58.4 ± 2.0 | 1260 ± 455 |
| anti-Bg | 7.3 ± 1.8 | 102 ± 23 | 40 ± 10 | 24 ± 4 | 101 ± 32 | 59.0 ± 2.2 | 1110 ± 332 |
| anti-Bn | 8.6 ± 3.1 | 103 ± 27 | 64 ± 26 | 40 ± 22 | 109 ± 12 | 59 3 ± 2.4 | 1020 ± 115 |
| anti-Br | 7.0 ± 0.9 | 87 ± 20 | 75 ± 20 | 33 ± 12 | 120 ± 38 | 58.5 ± 2.7 | 1379 ± 247 |
| anti-Ec | 7.9 ± 1.3 | 99 ± 16 | 54 ± 26 | 88 ± 64 | 102 ± 22 | 44.3 ± 3.6 | 2456 ± 1368 |
| anti-El | 7.5 ± 0.6 | 104 ± 25 | 47 ± 19 | 82 ± 50 | 86 ± 23 | 40.6 ± 0.5 | 1967 ± 660 |
| anti-Eo | 9.0 ± 1.5 | 117 ± 30 | 66 ± 32 | 97 ± 57 | 86 ± 29 | 38.5 ± 2.7 | 2771 ± 1122 |
| anti-Ep | 8.0 ± 0.9 | 109 ± 18 | 82 ± 54 | 229 ± 134 | 119 ± 42 | 41.0 ± 1.3 | 3169 ± 474 |
| Control** | 5.7 ± 0.6 | 82 ± 3 | 49 ± 9 | 24 ± 6 | 80 ± 53 | 38.0 ± 3.8 | 1137 ± 654 |
| Reference*** | 1.3-4.2 | 12-147 | --- | --- | 17-192 | --- | 218-2705 |

ALT: Alanine aminotransferase; AST: Aspartate aminotransferase; ALP: Alkaline phosphatase; CK: Creatine kinase.

*Baa: *Bitis arietans arietans*; Bg: *B. gabonica*; Bn: *B. nasicornis*; Br: *B. rhinoceros*; Ec: *Echis c*oloratus; El: *E. leucogaster*; Eo: *E. ocellatus*; Ep: *E. pyramidum*

**Control refers to a group of non-immunized rabbits.

***Reference range for New Zealand rabbits [1].

Table C: Cross-reactivity by ELISA of anti-*Bitis* spp. sera*.

| Antiserum | Venom | | | |
| --- | --- | --- | --- | --- |
|  | *B. a. arietans* | *B. gabonica* | *B. nasicornis* | *B. rhinoceros* |
| anti-*B. a. arietans* | 110 ± 59 | 29 ± 16 | 22 ± 10 | 25 ± 13 |
| anti-*B. gabonica* | 64 ± 36 | 106 ± 36 | 40 ± 18 | 73 ± 46 |
| anti-*B. nasicornis* | 43 ± 18 | 60 ± 11 | 94 ± 15 | 38 ± 3 |
| anti-*B. rhinoceros* | 81 ± 38 | 104 ± 31 | 65 ± 32 | 97 ± 40 |

*Results correspond to the relative concentration of specific antibodies expressed as percentage, 100% corresponding to the titer of serum raised against the homologous venom of each species. Results are expressed as mean ± SD of all rabbits in each group (n = 4).

Table D: Cross-neutralization of lethality* of venoms of African *Bitis* species.

| Antiserum | Venom | | | |
| --- | --- | --- | --- | --- |
|  | *B. a. arietans* | *B. gabonica* | *B. nasicornis* | *B. rhinoceros* |
| anti-*B. a. arietans* | 0.6  (0.2-1.7) | NA** | NA | NA |
| anti-*B. gabonica* | 0.5  (0.3-0.9) | 0.7  (0.4-1.3) | 1.0  (0.6-1.7) | 0.5  (0.3-0.8) |
| anti-*B. nasicornis* | 0.6  (0.3-1.4) | 0.6  (0.3-0.9) | 2.6  (1.6-4.0) | 0.3  (0.1-0.6) |
| anti-*B. rhinoceros* | 0.6  (0.4-1.0) | 0.6  (0.3-0.9) | 0.3  (0.1-0.6) | 1.1  (0.7-1.9) |

*Neutralizing ability of pools of serum samples from all rabbits in each group is expressed as ED_50_ (i.e., the ratio mg venom/mL antiserum at which the lethality of the venoms was reduced to 50%). Values in parenthesis correspond to the 95% confidence intervals.

**NA: Analysis not done, since the antiserum showed reactivities by ELISA lower than the pre-established limit of 33% against heterologous venoms, which was selected as our threshold, and therefore it was not assessed in the *in vivo* neutralization assays to avoid the unnecessary use of mice.

Table E: Cross-neutralization of hemorrhagic activity* of venoms of African *Bitis* species.

| Antiserum | Venom | | | |
| --- | --- | --- | --- | --- |
|  | *B. a. arietans* | *B. gabonica* | *B. nasicornis* | *B. rhinoceros* |
| anti-*B. a. arietans* | 0.50 ± 0.11 | NA** | NA | NA |
| anti-*B. gabonica* | 0.42 ± 0.08 | 0.66 ± 0.08 | 0.54 ± 0.12 | 0.47 ± 0.06 |
| anti-*B. nasicornis* | < 0.30*** | 0.35 ± 0.03 | 2.22 ± 0.42 | < 0.30 |
| anti-*B. rhinoceros* | 0.53 ± 0.13 | 0.94 ± 0.06 | < 0.30 | 1.13 ± 0.28 |

*Neutralizing ability of pools of serum samples from all rabbits in each group is expressed as ED_50_ (i.e., the ratio mg venom/mL antiserum at which the hemorrhagic activity of the venoms was reduced to 50%). Values corresponds to the mean ± SD (n = 3).

**NA: Analysis not done, since the antiserum showed reactivities by ELISA lower than the pre-established limit of 33% against heterologous venoms, which was selected as our threshold, and therefore it was not assessed in the *in vivo* neutralization assays to avoid the unnecessary use of mice.

***0.30 mg venom/mL antiserum is the lower ratio that the technique allowed to test. When no neutralizing activity was detected at this ratio, the result was reported as < 0.30.

Table F: Cross-reactivity by ELISA of anti-*Echis* spp. sera*.

| Antiserum | Venom | | | |
| --- | --- | --- | --- | --- |
|  | *E. coloratus* | *E. leucogaster* | *E. ocellatus* | *E. pyramidum* |
| anti-*E. coloratus* | 96 ± 36 | 63 ± 18 | 87 ± 14 | 99 ± 17 |
| anti-*E. leucogaster* | 97 ± 37 | 107 ± 30 | 96 ± 37 | 150 ± 33 |
| anti-*E. ocellatus* | 46 ± 9 | 39 ± 12 | 103 ± 30 | 80 ± 25 |
| anti-*E. pyramidum* | 53 ± 37 | 49 ± 29 | 47 ± 27 | 99 ± 48 |

*Results correspond to the relative concentration of specific antibodies expressed as percentage, 100% corresponding to the titer of serum raised against the homologous venom of each species. Results are expressed as mean ± SD of all rabbits in each group (n = 4).

Table G: Cross-neutralization of lethality* of venoms of African *Echis* species.

| Antiserum | Venom | | | |
| --- | --- | --- | --- | --- |
|  | *E. coloratus* | *E. leucogaster* | *E. ocellatus* | *E. pyramidum* |
| anti-*E. coloratus* | 1.8  (1.3-2.3) | 1.3  (1.1-1.9) | 0.6  (0.4-1.1) | 2.0  (1.4-3.3) |
| anti-*E. leucogaster* | 1.8  (1.3-2.5) | 2.9  (2.2-3.9) | 1.0  (0.7-1.6) | 2.5  (1.8-3.4) |
| anti-*E. ocellatus* | 0.9  (0.3-1.4) | 0.7  (0.3-1.0) | 1.2  (0.9-1.6) | 0.5  (0.4-0.8) |
| anti-*E. pyramidum* | 0.6  (0.3-0.8) | 0.9  (0.3-1.4) | 0.5  (0.4-0.8) | 1.7  (0.9-2.3) |

*Neutralizing ability of pools of serum samples from all rabbits in each group is expressed as ED_50_ (i.e., the ratio mg venom/mL antiserum at which the lethality of the venoms was reduced to 50%). Values in parenthesis correspond to the 95% confidence intervals.

Table H: Cross-neutralization of hemorrhagic activity* of venoms of African *Echis* species.

| Antiserum | Venom | | | |
| --- | --- | --- | --- | --- |
|  | *E. coloratus* | *E. leucogaster* | *E. ocellatus* | *E. pyramidum* |
| anti-*E. coloratus* | 1.32 ± 0.17 | 1.09 ± 0.60 | 1.26 ± 0.12 | 1.70 ± 0.88 |
| anti-*E. leucogaster* | 1.14 ± 0.28 | 2.00 ± 0.08 | 1.78 ± 0.36 | 2.26 ± 0.81 |
| anti-*E. ocellatus* | 0.35 ± 0.01 | 0.35 ± 0.04 | 1.36 ± 0.47 | 0.45 ± 0.08 |
| anti-*E. pyramidum* | 0.72 ± 0.23 | 1.24 ± 0.62 | 1.55 ± 0.44 | 1.59 ± 0.74 |

*Neutralizing ability of pools of serum samples from all rabbits in each group is expressed as ED_50_ (i.e., the ratio mg venom/mL antiserum at which the hemorrhagic activity of the venoms was reduced to 50%). Values corresponds to the mean ± SD (n = 3).

Table I: Cross-neutralization of coagulant activity* of venoms of African *Echis* species.

| Antiserum | Venom | | | |
| --- | --- | --- | --- | --- |
|  | *E. coloratus* | *E. leucogaster* | *E. ocellatus* | *E. pyramidum* |
| anti-*E. coloratus* | < 0.20** | 0.24 ± 0.00 | < 0.20 | 0.89 ± 0.10 |
| anti-*E. leucogaster* | < 0.20 | 0.60 ± 0.17 | < 0.20 | 0.74 ± 0.00 |
| anti-*E. ocellatus* | < 0.20 | < 0.20 | 0.32 ± 0.00 | < 0.20 |
| anti-*E. pyramidum* | < 0.20 | < 0.20 | < 0.20 | 1.14 ± 0.00 |

*Neutralizing ability of pools of serum samples from all rabbits in each group is expressed as ED (i.e., the ratio mg venom/mL antiserum at which the change in absorbance is prolonged three times as compared to plasma incubated with venom alone). Values corresponds to the mean ± SD (n = 3).

**0.20 mg venom/mL antiserum is the lower ratio that the technique allowed to test. When no neutralizing activity was detected at this ratio, the result was reported as < 0.20.

**Reference**

1. Hewitt CD, Innes DJ, Savory J, Wills MR. Normal biochemical and hematological values in New Zealand white rabbits. Clin Chem. 1989; 35(8):1777-9. PMID: 2758652.
